# Exploring the tumor micro-environment in primary and metastatic tumors of different ovarian cancer histotypes

**DOI:** 10.3389/fcell.2023.1297219

**Published:** 2024-01-24

**Authors:** Bingqing Xie, Susan Olalekan, Rebecca Back, Naa Asheley Ashitey, Heather Eckart, Anindita Basu

**Affiliations:** Section of Genetic Medicine, Department of Medicine, University of Chicago, Chicago, IL, United States

**Keywords:** single-cell tumor profiling, ovarian cancer histotypes, primary tumor site, metastatic tumor site, ligand-receptor analysis, cell type annotation, cancer associated fibroblasts, tumor micro-environment

## Abstract

Ovarian cancer is a highly heterogeneous disease consisting of at least five different histological subtypes with varying clinical features, cells of origin, molecular composition, risk factors, and treatments. While most single-cell studies have focused on High grade serous ovarian cancer, a comprehensive landscape of the constituent cell types and their interactions within the tumor microenvironment are yet to be established in the different ovarian cancer histotypes. Further characterization of tumor progression, metastasis, and various histotypes are also needed to connect molecular signatures to pathological grading for personalized diagnosis and tailored treatment. In this study, we leveraged high-resolution single-cell RNA sequencing technology to elucidate the cellular compositions on 21 solid tumor samples collected from 12 patients with six ovarian cancer histotypes and both primary (ovaries) and metastatic (omentum, rectum) sites. The diverse collection allowed us to deconstruct the histotypes and tumor site-specific expression patterns of cells in the tumor, and identify key marker genes and ligand-receptor pairs that are active in the ovarian tumor microenvironment. Our findings can be used in improving precision disease stratification and optimizing treatment options.

## 1 Introduction

Ovarian cancer is the second most common and most malignant cancer in the female reproductive tract. Epithelial ovarian cancers (EOC) which account for about 90% of ovarian malignancies can be further divided into serous, endometrioid, clear cell, and mucinous histotypes (Matulonis et al., 2016). The risk factors of epithelial ovarian cancer vary by histotype but generally include age, weight, hormone therapy after menopause, as well as family history (American Cancer Society, 2020). Previous genomic studies (Network, 2011) have demonstrated that mutations in TP53 and NF1, and dysfunction of BRCA1 are involved in the pathogenesis of the serous carcinoma in the ovary (Sangha et al., 2008). However, only a few studies have investigated the cellular landscape and transcriptomic profile of ovarian cancer histotypes which can inform targeted therapies. In recent years, the development of single-cell technology allows researchers to zoom in on the cell-level transcriptome of the tumor tissue and provides a better understanding of the tumor microenvironment (TME).

Single-cell technology has been applied to ovarian cancer previously on malignant abdominal fluid (ascites) associated with high grade serous ovarian carcinoma (HGSOC) histotype (Izar et al., 2020) to resolve the HGSOC landscape and investigate inflammation programs. The stress associated chemo-resistance in solid tumors from metastatic sites with HGSOC was investigated together with stroma signaling to provide insight into chemotherapy resistance (Zhang et al., 2022). A recent study used scRNA-seq on primary and untreated peritoneal metastatic sites (Kan et al., 2022) and identified a subset of RGS5+ cancer-associated fibroblasts (CAFs) strongly supporting tumor metastasis and cancer recurrence in EOC. However, comparisons across multiple sites and histotypes are yet to be performed. We previously reported the cellular composition of metastatic ovarian tumors from the omentum using single-cell RNA sequencing technology, categorized tumor samples based on T cell infiltration signatures which were confirmed by immunohistochemistry, and identified the presence of a GNLY + CD4 T cell population in high T cell infiltrated samples (Olalekan et al., 2021).

In this current study, we characterized primary and metastatic tumors of different histotypes from 12 ovarian cancer patients using Drop-seq, a high-throughput single-cell RNA-seq technique (Evan et al., 2015). We analyzed the distribution of cell types with the tumor microenvironment and investigated possible cell-cell interactions by histotype. Cancer stem cells with increased expression of *IFIT1, IFIT2, IFIT3,* and *ISG15* were uniquely present in HGSOC and MMMT tumors. Cancer-associated fibroblast (CAF) sub-clusters showing high expression of *IL6, CCL2, S100A4, PDPN*, and *FGF7* were identified in both primary and metastatic samples. Our previously reported immune cell populations were validated in this bigger sample collection. Finally, we identified a cluster of *IL32*+ plasma B cells that were found exclusively in the primary tumor sites that may be of prognostic value.

With the inclusion of additional histotypes and tumor sites in our collection, this study allows us to characterize the differences in cell compositions between sites and different levels of their T cell infiltration, build cell or gene signatures to characterize the different ovarian cancer histotypes, and further investigate the underlying molecular mechanism in the TME. We further explored cell-to-cell communication among different cell sub-clusters, using inferred ligand-receptor (LR) interactions. We note that such interactions are enriched among epithelial cells and fibroblasts, and that LR interaction signatures vary across different tumor sites and histotypes.

## 2 Results

### 2.1 Establishing cell lineages, molecular subtypes, and cell-cycle states across samples

**FIGURE 1:**
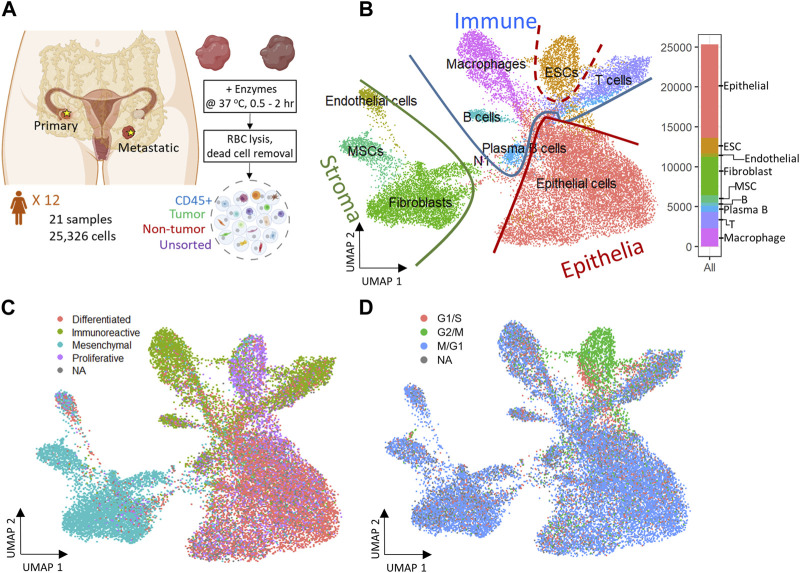
Experiment design and 2D reduced representation of all cells included in the study, annotated by major cell lineage, predicted cancer subtype, and cell cycle phase. **(A)** Profiling ovarian cancer tumor samples of different using droplet single cell RNA-seq. **(B)** All cell types projected on UMAP divided by Epithelia, Immune, and Stroma subpopulations. **(C)** Predicted cancer subtype projected on UMAP. **(D)** Cell cycle assignment projected on UMAP.

To study the cell composition of ovarian cancers, tumor tissues resected from 12 ovarian cancer patients undergoing debulking surgery in the ovaries, omentum, and rectum were analyzed (Figure 1A; Table 1). Briefly, the cohort consisted of seven white, two Asian, two Black, and one woman of unknown racial origin, and ranged between 39–77 years in age (mean ∼62 years). Most patient tumors were stage IIIB or above, according to staging by a pathologist. Solid tumor samples of different histotypes were collected from primary (ovaries) and metastatic (omentum, rectum) sites (Table 2) which enabled us to investigate histotype- and site-specific signatures at single cell level. Tumor samples were obtained fresh from surgery and processed using Drop-seq (Evan et al., 2015) within 24 h or fixed in formalin for immunohistochemistry (IHC). Immune (CD45^+^) and cancer cells enriched from a subset of samples were also profiled by Drop-seq to obtain a better representation of immune cells in our single-cell data.

**TABLE 1:** Metadata for ovarian cancer patients included in our study.

Patient	Histotype	Total/CD45/Tumor enrich			Pathological stage	Age	Race	Neo-adjuvant
P1	Malignant mixed Müllerian tumor (MMMT)	Yes	Yes	No	ypT3c, NX/yIIIC	66	Asian	Yes
P2	Clear cell	Yes	Yes	Yes	pT3c NX/IIIC	74	White	No
P3	Endometrioid with serous features	No	Yes	No	pT3b, N0/IIIB	66	White	No
P4	Endometrioid and serous features	No	Yes	No	pT1a N0/IA	69	White	No
P5	Clear cell	Yes	Yes	No	pT2B, N0/IIB	77	White	No
P6	High grade serous ovarian carcinoma (HGSOC)	Yes	No	No	pT3c Nx Mx/IIIc	56	White	No
P7	Serous ovarian carcinoma (SOC)	Yes	Yes	Yes	ypT3a Nx M1/Ivb	62	White	Yes
P8	High grade serous ovarian carcinoma (HGSOC)	Yes	No	No	pT3c N0/IIIC	48	UNK	No
P9	Low grade serous ovarian carcinoma (LGSOC)	Yes	No	No	pT3c, NX, M1b/IVB	39	White	No
P10	High grade serous ovarian carcinoma (HGSOC)	Yes	No	No	ypT3c N1a/yIIIc	66	Black	Yes
P11	High grade serous ovarian carcinoma (HGSOC)	Yes	No	No	pT3c Nx/IIIc	46	Asian	No
P12	High grade serous ovarian carcinoma (HGSOC)	Yes	No	No	pT3c, N1, M1/IIIc	71	Black	Yes

**TABLE 2:** Total number of cells and breakdown of cellular composition in each sample, color-coded by tumor site (Primary, Meta) and T cell infiltration (T_inf_) status.

Sample			Drop-seq										IHC			Category
Patient	Sample	Site	# cells	Epithelial cell (%)	ESC (%)	Endothelial cell (%)	Fibroblast (%)	MSC (%)	Macrophage (%)	B cell (%)	Plasma B cell (%)	T cell (%)	CK7 (%)	VIM (%)	CD45 (%)	Stage Tinf
1	P1-1	Omentum	2,227	59.5	4.5	1.1	22.9	3.6	4.5	0.6	1	2.3	59.2	60	7.9	Meta Low
	P1-2	Left Ovary	1,156	66.2	3.7	2.7	21.8	2.6	1.1	0	1	1	68.7	37.5	0.4	Primary Low
	P1-3	Right Ovary	1,051	76.6	5.3	0.9	10.1	3.4	0.9	0	0.7	2.2	66.6	50.2	6.1	Primary Low
2	P2-1	Omentum	1,008	54.6	8.1	1	8.7	1.7	10.6	0	2.9	12.3	66.6	79.4	58.9	Meta High
	P2-2	Right Ovary	330	28.2	6.1	0.6	21.8	3.9	6.4	0	10.9	22.1	89.3	70.6	34	Primary High
3	P3-1	Rectum	345	26.6	2.4	1.7	27.1	20.4	16.4	0.1	0.3	5.1	69.8	37.9	4.4	Meta Low
	P3-2	Left Ovary	928	42.3	3.8	3.8	24.9	14.5	9	0	0	1.7	70.7	40.5	0.7	Primary Low
4	P4-1	Left Ovary	837	37.2	9.2	1.2	9.4	2.5	35.6	0.1	0.6	4.2	76.4	55.3	0.8	Primary Low
5	P5-1	Right Ovary	2,554	45.7	11.4	0.4	18.7	2.4	5.6	0	3.7	12.1	84.6	54	17.8	Primary High
6	P6-1	Omentum	3,357	28.3	6.5	1	22.6	2	9.1	8.8	3.8	17.4	57.5	54.6	17.3	Meta High
	P6-2	Left Ovary	2,102	60.7	15.2	1.6	11.5	4.8	2.6	0	0.3	2.6	69.1	34.4	9.8	Primary Low
7	P7-1	Omentum	3,542	43.7	5.6	4	10.9	1.5	12.1	2.1	7.7	12.5	41.7	69.1	28.5	Meta High
8	P8-1	Right Ovary	359	70.2	9.6	1.9	5.8	2.1	5.4	0	0.6	4.3	83	39.2	3.3	Primary Low
	P8-2	Left Ovary	467	55.7	7.2	3.9	5.9	6.4	11.7	0	0.6	8.6	85.5	51.2	2.5	Primary Low
9	P9-1	Omentum	529	32.9	2.8	2.8	19.1	5.1	16.6	0.2	5.7	14.7	38.9	48	4.4	Meta High
	P9-2	Omentum	585	35.6	2.1	3.1	40.9	4.4	8	0	3.4	2.6	50.6	49.4	5.4	Meta Low
	P9-3	Left Ovary	341	38.7	1.8	0	36.1	10.3	9.4	0	1.2	2.4	84.2	34	2.5	Primary Low
	P9-4	Right Ovary	378	30.2	1.9	1.9	38.6	9.3	13.8	0	0	4.2	78.1	41.7	2.3	Primary Low
10	P10-1	Omentum	1,067	27.7	8.1	2.4	34.3	1.6	17.2	1	2.2	5.5	21	59.3	10.7	Meta Low
11	P11-1	Omentum	1,066	56.9	21.2	0.2	2.9	0.4	10.4	0.3	2.5	5.2	57.8	77.9	23.7	Meta Low
12	P12-1	Omentum	1,097	43.3	5.6	4.4	37.4	5.3	1.3	0.5	1	1.2	78.8	44.4	4	Meta Low

The cell types are abbreviated as follows: EP, Epithelial cells; TC, T cells; MA, Macrophages; EN, Endothelial cells; BC, B cells; FB, Fibroblasts; MS, Mesenchymal stem cells; ES, Embryonic stem cells.

A total of 26 gene expression matrices were generated from Drop-seq experiments on 21 ovarian cancer tumors from 12 patients. We identified a total of 38,811 genetic features across 25,326 cells from tumors resected from multiple tissue sites in this study. The filtered gene expression matrices were integrated using the anchor-based alignment. Unsupervised clustering analysis yielded 11 distinct clusters of cells. The resulting clusters were annotated using Template-based Automated Cell type Assignment (sc-TACA; Section 4), yielding ten major cell types including epithelial, endothelial, mesenchymal stem (MSC), embryonic stem (ESC), fibroblast, macrophage, T, B, and plasma B cells and a small cluster of 37 cells marked as N1 that shared markers with astrocytes which we saw in our previous study (Olalekan et al., 2021) (Figure 1B). Percentages of each cell type comprising each tumor sample are shown in Supplementary Figure S1A; Table 2. Due to the small number of N1 cells in any given sample (<0.1%), we excluded them from further analysis. For simplicity, the cell types were classified into three compartments: *epithelia,* containing epithelial cells and ESC, *stroma* containing endothelial cells, MSC and fibroblasts, and *immune*, containing macrophages, B and plasma B cells, and T cells (Figure 1B).

Next, we explored the expression of the genes associated with the four molecular subtypes of ovarian cancer - differentiated, immunoreactive, mesenchymal, and proliferative - identified from The Cancer Genome Atlas (TCGA) (Network, 2011) in our dataset. We were able to assign one of the four molecular subtypes with the highest TCGA module score to 93.7% of cells; cells with a negative module score were marked as not assigned (NA) (Olalekan et al., 2021). When each cell on the dimension reduction of the Uniform Manifold Approximation and Projection (UMAP) was marked with the molecular subtype assigned to it (Figure 1C), we noted that the major cell types and the cellular compartments they belong to (Figure 1B) match the predominant molecular subtype of ovarian cancer identified by TCGA. The epithelial cells were distributed through all four cancer subtypes and comprised 80% of the cells predicted as differentiated subtype. 73% of cells from the predicted immunoreactive subtype were immune cells (B cells, T cells, and macrophages). The mesenchymal subtype, associated with worst survival (Konecny et al., 2014), consisted of the least epithelial cells and contained the highest percentage (82%) of stroma cells, including MSC, fibroblasts, and endothelial cells. The proliferative subtype contained 56% of cells from the epithelial cell category; 26.2% of cells from the ESC (about half of the total ESC population) that also showed unique stem cell features described later, were of the proliferative subtype. Sample-specific composition of TCGA subtypes is shown in Supplementary Figure S1B.

To study the cell cycling effects under the TME, Cell cycle analysis was performed on the combined dataset to assign a cell-cycle module score to each cell for the G1/S, G2/M, and M/G1 phases. Cells that could not be assigned to one of these phases were marked as “NA”. We noted that the cell cycling patterns were roughly similar for all cell types (Figure 1D), with the exception of ESC. A large fraction of cells across all cell types were assigned to the M/G1 phase (64.3%; Supplementary Figure S1C), as seen previously (Tay et al., 1991). In contrast, most ESC (>70%) were assigned to the G2/M phase where they likely stalled during the cell cycle (Becker et al., 2006).

### 2.2 Immune cells and their expression in ovarian cancer samples

We identified 5,453 cells as immune cells that could be further split into B cells, plasma B cells, T cells, and macrophages (Supplementary Figure S2A). We also found a few dendritic cells and common myeloid progenitor cells (n = 52 and 30, respectively) that co-clustered with macrophages; these cells were not included in the downstream analysis due to the low cell counts. When identifying the subclusters within each cell type, we denote them as “EP” for epithelial cells, “TC” for T cells, “BC” for B cells, “MA” for macrophages, “ES” for ESC, “FB” for fibroblasts, “MS” for MSC, and “EN” for endothelial cells. We used a single digit starting from 0 to index the sub-clusters for each cell type, e.g., EP0 denotes cluster 0 of epithelial cells. An adjusted *p*-value threshold (adjusted *p* < 0.05) was applied to all the gene markers mentioned below for sub-clusters.

**FIGURE 2:**
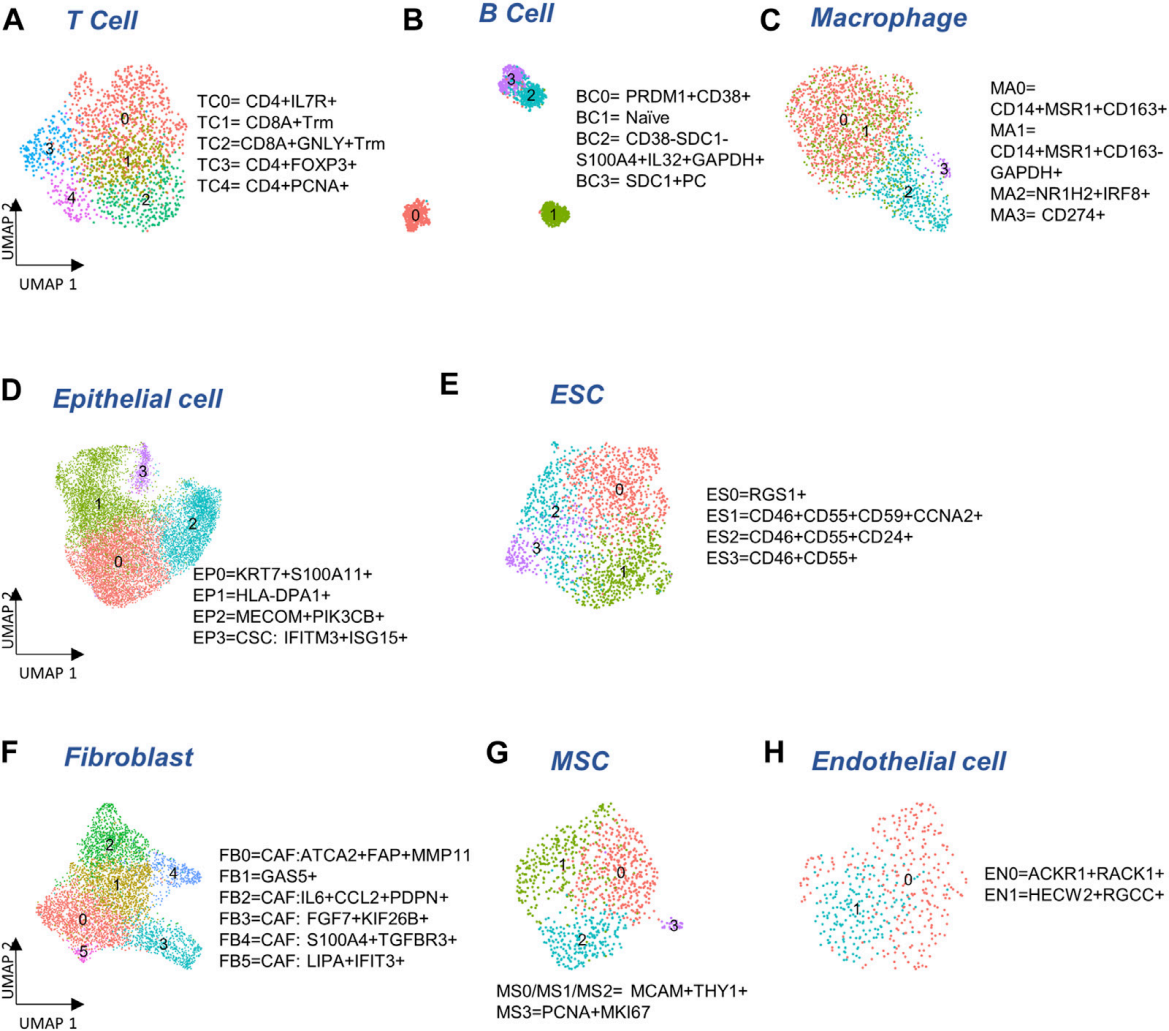
Cellular sub-types in the Immune, Epithelia, and Stroma. **(A–C)** Subclusters of major immune cell types: T cells, B cells, and macrophages, respectively. **(D,E)** Subcluster annotation for epithelial and embryonic stem cells (ESC), respectively. **(F–H)** Subcluster annotation for major cell-types in the stroma: fibroblasts, mesenchymal stem cells, and endothelial cells, respectively.

To determine if there were any cells unique to the different tumor sites, we cross-referenced 5,371 immune cells with our previous study (Olalekan et al., 2021) of metastatic ovarian tumors. We identified five subclusters (Figure 2A), consisting of three clusters of CD4^+^ cells (TC0, TC3, TC4), and two clusters of CD8^+^ (*CD8A,* logFC >0.7) resident memory T (T_rm_) cells (TC1 and TC2). Among these clusters, one subcluster containing GNLY+CD8+T_rm_ (*GNLY,* logFC = 1.9) cells (TC2) that was not observed previously (Olalekan et al., 2021), derived from a subset of metastatic samples, P5-1, P6-1, P7-1. Three CD19-PRDM1+JCHAIN + plasma B clusters, BC0, BC2, and BC3, and one naïve B cluster, BC1 expressing *CD19* (logFC = 1.4) were observed (Figure 2B; Supplementary Figure S2B). The BC2 subcluster that consisted of CD38-SDC1-S100A4+IL32+GAPDH+ (logFC >2.1) plasma B cells, has not been identified previously (Olalekan et al., 2021). The marker *IL32* (logFC = 2.6) was a proliferation marker for malignant plasma cells in myeloma (Aass et al., 2022). Intriguingly, we find BC1 to be almost absent in the primary tumor site (ovary). Four macrophage subclusters (Figure 2C; Supplementary Figure S2B) were annotated, including a CD14+MSR1+CD163-cluster (MA1) that were mainly found in samples collected from the primary tumor site (ovary) and thus not seen in our previous study (Olalekan et al., 2021).

### 2.3 Epithelial cells and their expression in ovarian cancer samples

We detected 11,716 epithelial cells comprising the *epithelia*, as the most abundant cell type in our integrated and batch-corrected dataset. Hierarchical clustering of these cells (resolution = 0.3) detected four epithelial sub-clusters, EP0-3 (Figure 2D). Dot plots for some top differentially expressed markers, *EPCAM, S100A11, KIAA1217, MAML2, MECOM, IFIT2/3, and LIPA* are shown in Supplementary Figure S2C. The EP0 sub-cluster comprised 38% of all epithelial cells and showed a distinctive signature of cytokeratin genes, *KRT19* (logFC = 1), *KRT18* (logFC = 0.8), and *KRT7* (logFC = 0.6) (Supplementary Table S1A). A recent study on the origin of ovarian cancer (Hu et al., 2020) connected fallopian tube epithelial cell subtypes to intra-tumor heterogeneity in serous ovarian cancer (SOC), and used *KRT7* as a marker for secretory epithelial (SE) cells in the fallopian tube as the cell-of-origin for SOC. Other genes found upregulated in EP0 (Supplementary Table S1A) were *S100A6* (logFC = 0.8) and *S100A11* (logFC = 0.7) from the S100 calcium-binding protein family. The S100 protein family interacts with cytoskeletal proteins (Schneider et al., 2008), and may promote metastasis and stimulate angiogenesis. Specifically, the *S100A11* gene (Cui et al., 2021) acts as a tumor promoter by regulating MMP activity and the epithelial-mesenchymal transition (EMT) process. Another top expressing marker gene, *LGALS3* (logFC = 0.8) is associated with cell migration, proliferation, adhesion, and cell-cell interaction in tumor cells, and is implicated in tumor progression and chemo-resistance of epithelial ovarian cancer (Wang et al., 2019). EP1 cluster exhibited significant upregulation of genes belonging to the MHC class II protein family, *HLA-DPA1*, *HLA-DRA*, *HLA-DPB1*, and *HLA-DRB1* (logFC >1), was associated with the KRT17 sub-cluster of secretory epithelial cells in the fallopian tube epithelia (Kim et al., 2012) as well as high expression of ribosomal proteins such as *RPLP1* (logFC = 0.8) and *RPS6* (logFC = 0.8). EP2 subcluster highly expressed markers of *SOS1* (logFC = 0.7), *MAPK8* (logFC = 0.8), and *PIK3CB* (logFC = 0.6) where those markers were found in enriched chromatin pathways, growth factor signaling pathways, such as platelet-derived growth factor (PDGF), nerve growth factor (NGF), epidermal growth factor receptor (EGFR), platelet-derived growth factor receptor Beta (PDGFRB), and angiopoietin like protein 8 (ANGPTL8) regulatory pathways. Protein families with ankyrin-repeat proteins *ANK* (logFC >0.3) and zinc finger proteins associated with cancer progression (Scurr et al., 2008; Jen and Wang, 2016) were upregulated in EP2. EP3 showed a unique signature of interferon-stimulated genes *IFIT1-3* (logFC >2.5), *IFITM1-3* (logFC >0.6), and *ISG15* (logFC = 2.5), previously characterized as markers of cancer stem cells (CSC) (Behera et al., 2020). Detailed marker information is provided in Supplementary Table S1A.

We also detected 1,925 embryonic stem cells (ESC) in our combined dataset that showed moderate expression of ESC markers, *STAT3* and *CTNNB1* (Supplementary Figure S2D). Further clustering of ESC yielded 4 sub-clusters (Figure 2E): ES0 exhibited markers of the immunoreactive molecular subtype, such as *RGS1* (Hu et al., 2021) (logFC = 1.9), *CD3E* (Barber et al., 1989) (logFC = 0.6), and *CD3G* (Barber et al., 1989) (logFC = 0.8); also see Supplementary Table S1C. We found cancer stem cell gene, *CD24* (Li et al., 2017) (logFC = 0.9), and therapy resistant genes, *CD46* (logFC = 0.3) and *CD55* (Zhou et al., 2008) (logFC = 0.4) expressed in ES1, ES2, and ES3 combined, compared to ES0, and cancer stem cell marker, *CD59* (Chen et al., 2017) to be the highest in ES1 (logFC = 0.4, Supplementary Figure S2D). Analysis of cell cycle activity (Figure 1D) assigned 73% of the ESC to the G2/M and 22.5% to the G1/S phases. The prevalence of cells arresting in the G2/M phase has been previously associated with cancer cell proliferation, mutation of *TP53* and *KARS*, T cell infiltration, and cancer metastasis (Oshi et al., 2020a; Oshi et al., 2020b; Jung et al., 2021). Moreover, the expression of CDKN1A and senescence gene FN1 with the lack of expression of PCNA can trigger the G2 arrest or the stress-induced premature senescence (SIPS) found in a previous cancer study (Mirzayans et al., 2012) (Supplementary Figure S2D). Meanwhile the shortened G1 phase regulated by *TP53* can lead to DNA damage, and subsequently affect the S phase with malfunctioned G1/S checkpoint (Viner-Breuer et al., 2019). Low number of cells in MG1 (<5%) may indicate the ESC to be post-mitotic. Specifically, genes expressed in ES1 are enriched for cell cycle functions and G2/M transition (Supplementary Table S1D). Increased gene expression required for G2/M transition and indicative expression for DNA damage response, such as *CCNA2, CCNB1, CCNB2, CDK1, CKAP5, DCTN3,* and *TUBB4B* can support the malfunction of P53 (Taylor and Stark, 2001) (Supplementary Figure S2D).

### 2.4 Stromal cells and their expression in ovarian cancer samples

The *stroma* compartment contained three major subsets: fibroblasts, MSC and endothelial cells. Hierarchical clustering of 4,772 fibroblasts (resolution = 0.5) yielded six sub-clusters (Figure 2F) where five of them contain markers for cancer-associated fibroblasts (CAF). The CAF-like clusters involved multiple molecular mechanisms associated with tumor progression, angiogenesis via vascular endothelial growth factor A (*VEGFA*) production, and coordination of immune function through chemokine and cytokine (Kalluri, 2016) production. FB0 and FB1 showed comparatively high expression of myofibroblast markers, *ACTA2* (logFC = 1.0) and *MYL9* (Liu et al., 2019) (logFC = 0.7). CAF associated markers, *MMP11*, *MMP2*, *FAP*, *THY1*, *IFI6*, *IFI27* (Puram et al., 2017) (logFC >0.3) were highly expressed in FB0 (Supplementary Figure S2E; Supplementary Table S2A). In contrast, we did not find any CAF-related expression in FB1. FB2 showed upregulation of NF-kappa B signaling pathway genes, *NFKBIA*, *NFKB1*, and *NFKBIZ* (logFC >0.4), VEGFA-VEGFR2 signaling pathway gene, *VEGFA* (logFC = 0.3), chemokine receptor genes, *IL6* (logFC = 1.7) and *CCL2* (logFC = 1.7), transmembrane glycoprotein genes, *PDPN* (Neri et al., 2015) (logFC = 0.1), and genes associated with cancer metastasis, *IER3* (Ye et al., 2018) (logFC = 0.6), *SGK1* (Sang et al., 2021) (logFC = 1), and *SERPINE2* (Yang et al., 2018) (logFC = 0.5). Genes overexpressed in FB3 subcluster were enriched for angiogenesis, integrin signaling, and related to extracellular matrix remodeling, including *FGF7* (logFC = 1.2) and *S100A4* (logFC = 0.9). The FB4 subcluster exhibited elevated expressions of growth factor binding genes, *IGFBP4* (logFC = 0.8), *TGFBR3* (logFC = 0.8), and top markers *APOLD1* (logFC = 0.3) and *PLXND1* (logFC = 0.3) for angiogenesis and blood vessel development (Supplementary Tables S2A, 2B). FB5 showed upregulated genes highly enriched in immune crosstalk and cytokine/interferon signaling pathways. Particularly, interferon inducible genes, such as *IFI6*, *IFI27*, *IFI44*, *IFI44L*, *IFIH1*, and *IFIT1-3* (logFC >1.5) were highly expressed in FB5 the subcluster that might be due to the inflammatory crosstalk in the TME (Provance and Lewis-Wambi, 2019) (Supplementary Tables S2A, 2B). Taken together, all fibroblast subclusters exhibited CAF features, with the exception of FB1.

The progenitors of the stroma sub-population, 951 mesenchymal stem cells (MSC) were detected in our data that could be clustered into 4 sub-clusters (Figure 2G). The majority of the MSC (MS0-3) expressed MSC markers, *MCAM* and *THY1* (Figure 2G; Supplementary Figure S2F). A small subset of MSC (MS3) also expressed cycling markers *MKI67* (logFC = 2.1) and *PCNA* (logFC = 1.9) that was not seen in the other clusters (Supplementary Figure S2F).

Finally, a distinct population of 472 endothelial cells was found in the stromal compartment. Two sub-clusters, EN0 and EN1, both expressing endothelial markers, *ENG*, *S100A6*, and *CD34* (Middleton et al., 2005; Bao et al., 2012; Liu et al., 2014) were found (Figure 2H). EN0 showed higher expression of *ACKR1* (logFC = 1.2), carcinoma-associated genes *RACK1* (Berns et al., 2000) (logFC = 0.7) and *CD74* (Maharshak et al., 2010) (logFC = 0.8) (Supplementary Figure S2G; Supplementary Table S3A). *ACKR1* is associated with ligand transcytosis (Salazar and Zabel, 2019) and serves as a non-specific and promiscuous receptor for several inflammatory chemokines when expressed in endothelial cells (Peiper et al., 1995; Lee et al., 2003; Middleton et al., 2005). Genes upregulated in EN1 are related to angiogenesis and blood vessel morphogenesis in tumor metastasis (Supplementary Table S3B).

### 2.5 Cellular composition by ovarian cancer histotypes and tumor sites

**FIGURE 3:**
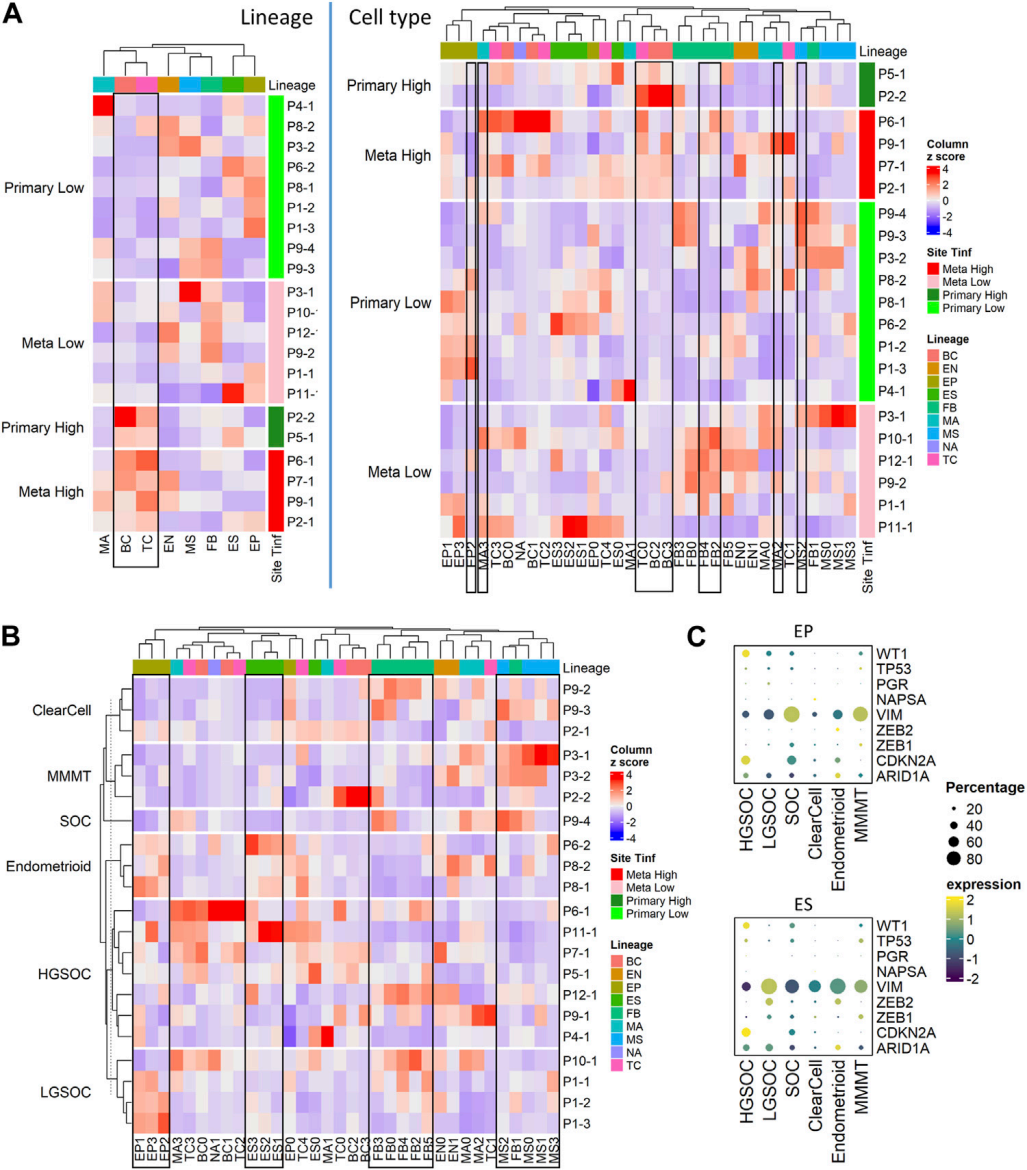
Cell composition by tumor site, T cell infiltration, and histotypes; fractions of immune, stromal, and epithelial cells are explored using immunohistochemistry. **(A)** Heatmap of major cell type composition (left) and sub cell type (right) for all patient samples. The column z-scores are calculated from cellular compositional percentages within each sample; the rows are split by site and T cell infiltration status. **(B)** Heatmap of cell type subclusters composition percentage for all patient samples. The values are column z-scores normalizing the percentage and the rows are split by histotypes. **(C)** Dot plot of histotype markers expression in epithelial (EP) and embryonic stem (ES) cells. The expression in the dot plot is the averaged scaled log normalized TP10k value.

We conducted further analysis on our tumor samples to examine cell types described above (Figure 1B; Supplementary Table S4A), cancer histotypes (Figure 3B; Table 1), and T cell infiltration into tumors (Figure 3A; Supplementary Figure S2A; Table 2). Based on pathology grading, the samples in this study belong to six ovarian cancer histotypes: serous ovarian carcinoma (SOC), high grade serous ovarian carcinoma (HGSOC), low grade serous ovarian carcinoma (LGSOC), clear cell, endometrioid with serous features, and malignant mixed Müllerian tumors (MMMT). Figure 3B shows the heatmap of cell type compositions combined across all samples, grouped by cancer histotypes. We noted the highest fraction of epithelial cells in MMMT and the highest fraction of MSC in endometrioid samples. Expression of previously established immunohistochemical markers (Köbel et al., 2016; Peres et al., 2018) *WT1*, *NAPSA*, and *PGR* for histotype classification were checked on EP and ES cell lineages. We confirmed higher expression of *WT1* in HGSOC, and *NAPSA* in clear cell histotypes compared to the remaining histotypes, and the presence of *PGR* expression in endometrioid histotype with serous features (Figure 3C). Due to the limitation of 3’ scRNA-seq assays, we were not able to assess the abnormality for TP53 to differentiate HGSOC and LGSOC. Therefore, the over-expression pattern for marker *CDKN2A* was used to identify HGSOC. The expression of *TP53* exhibited low prevalence across all histotypes (Figure 3C; Supplementary Figure S3C). The expression levels of additional markers (Sakata et al., 2017; Peres et al., 2018; Leskela et al., 2019) *VIM* and *ARID1A* were used to distinguish other histotypes. The EMT repressors, zinc finger E-Box binding homeobox 1 and 2, (*ZEB2, ZEB1*) (Sakata et al., 2017; Leskela et al., 2019) related to endometrial carcinosarcoma, a mix of (epithelial) carcinoma, and (mesenchymal) sarcoma, were used to distinguish endometrioid and MMMT histotypes. In epithelial and ES cells, we observed *WT1+CDKN2A* + for HGSOC, and *WT1+CDKN2A-VIM +* for LGSOC (Figure 3C). Among the other histotypes, we find SOC to be *WT1+CDKN2A + VIM+*, clear cell to be *WT1-NAPSA+*, endometrioid to be *WT1-NAPSA-PGR + ZEB2+ARID1A+*, and MMMT to be *WT1+VIM + ZEB1+* in our limited sample space (Figure 3C).

We project the histotype-specific GWAS (genome wide association study) markers (Lengyel et al., 2022) from HGSOC, LGSOC, clear cell, Endometrioid, and Mucinous histotypes on both epithelial and stromal lineages using averaged expression values in the dot plot (Supplementary Figure S3D). The HGSOC specific markers were more commonly detected in HGSOC samples compared to the other histotypes (Supplementary Figure S3D, rectangle in the HGSOC panel); the LGSOC specific markers were exclusive to the LGSOC samples (Supplementary Figure S3D, rectangle in the LGSOC panel); more HGSOC and less LGSOC specific markers were detected in the SOC sample (Supplementary Figure S3D, rectangle boxes in the SOC panel). The expressions of clear cell-specific markers were not exclusive or elevated in the clear cell samples (Supplementary Figure S3D, rectangle in the clear cell panel), while the expression of endometrioid specific markers were not exclusive but elevated in the endometrioid samples (Supplementary Figure S3D, rectangle in the endometrioid panel). The MMMT samples exhibited a mixed signal from all markers from different histotypes’ GWAS genes as well as the borderline GWAS markers samples (Supplementary Figure S3D, rectangles in the MMMT panel).

The secretory and ciliated epithelial markers, and hallmark epithelial markers collected in a previous study (Lengyel et al., 2022) were projected to HGSOC, LGSOC, and SOC samples. The ciliated epithelial marker *CAPS* was only expressed in EP0. The secretory epithelial marker *PAX8* was detected across the epithelial (EP and ES) lineage in HGSOC and SOC but absent in EP1 and all ES cells in LGSOC samples (Supplementary Figure S3E, orange rectangle). The markers for cell cycle (*CDK4*) and MHC II cluster (*HLA-DQA1, HLA-DPA1*) were absent in HGSOC (Supplementary Figure S3E, green rectangle). The markers for cytokeratin, *KRT23* and epithelial stem cell, *CD44* were only found in LGSOC’s EP and ES subpopulation respectively (Supplementary Figure S3E, purple rectangle). On the contrary, the markers for chromatin remodeling and pan-epithelial were lower expressed in LGSOC (Supplementary Figure S3E, blue rectangle).

Tumors from the ovaries were considered primary tumor sites while the tumors from the omentum and rectum were categorized as metastatic (Table 2). We categorized each sample based on the level of T cell infiltration (T_Inf_) (Olalekan et al., 2021), and whether its tumor site was primary or metastatic, thus grouping our samples into 4 categories: Metastatic High (T_Inf_), Metastatic Low, Primary High, and Primary Low. We found no significant differences in the composition of major cell lineages between primary and metastatic sites. However, at the sub-cellular level, the ratios of FB4, FB2, MA3, and MA2 were higher in metastatic sites, while EP2 showed an opposite pattern (Supplementary Tables S4B, C). T-tests performed on the ratios of different cell lineages showed significantly higher fractions of TC and BC in the high T_Inf_ group, and MS was lower in T_Inf_ (Supplementary Table S4B). Zooming in, the TC0 and BC3 appear to drive these differences, while higher MS2 correlated with low T cell infiltration (Figure 3A; Supplementary Table S4C). The percentages of each immune cell type in these four categories are shown in Supplementary Figure S2A (bottom panel). We found FB sub-clusters, FB0, FB2, FB4, and FB5 expressing CAF markers to be enriched in samples classified as Metastatic Low (Supplementary Figure S3B), along with CSC (EP3 sub-cluster, Supplementary Figure S3B).

Overall, the cell sub-type fractions from the same main cell types were correlated with each other. For example, the fractions of subclusters by sample in EP, ES, and CAF sub types- FB0, FB2, FB3, FB4, and FB5 were similar to each other and thus clustered together (Figure 3B, rectangles). Interestingly, the only non-CAF subcluster of fibroblast, FB1 clustered with MSC (Figure 3B, rectangle).

Due to the limited number of samples available for all histotypes, we were unable to calculate the statistical significance of the cell cluster compositions. Nevertheless, several intriguing observations merit attention. The EP0 cluster was observed in all histotypes (Supplementary Table S4A). The fractions of EP1, EP2, and EP3 cells were higher in MMMT compared to the other histotypes, while the fraction of cells in ES1, ES2, and ES3 were higher in HGSOC (Supplementary Figure S3A). For clear cell histotype, the percentage of cells in TC0, BC2, and BC3 were higher than in other histotypes. The endometrioid histotype showed a high fraction of MSC, MA1, and FB1. The percentage of certain macrophage and T cell subtypes in LGSOC (MA0, MA2, and TC1) was higher than in other histotypes. The SOC sample of undetermined pathology grade appeared more similar to HGSOC from the primary site than LGSOC in terms of cell type composition (Supplementary Figure S3A). In the SOC sample, the human leukocyte antigen (HLA) genes have higher average expression compared to HGSOC samples. BC2 derived from tumors with high T cell infiltration and were identified primarily in clear cell and SOC histotypes (Supplementary Figures S3A, B).

Immunohistochemical staining of vimentin (VIM), CD45, and cytokeratin-7 (CK7) was also performed on tumor tissues from metastatic (Supplementary Figure S3F) and primary (Supplementary Figure S3G) tumors belonging to different ovarian cancer histotypes to investigate the fractions of the major cell lineages in these tumors. We correlated the percentage of each cellular subset in our combined dataset from 18 samples to the IHC results; three samples from patients P3 and P4 that were enriched for CD45^+^ cells alone for Drop-seq were excluded from this analysis. The percentage that stained for CD45 was well correlated (Pearson correlation = 0.51 and a significant 0.03 *p*-value estimation, Supplementary Figure S3H, left panel) with the *immune* population (MA, TC, and BC). The correlations between area staining for CD45 (IHC), and the percentage of T cells, B cells, and macrophages out of all cells are 0.59, 0.53, and 0.17 (not significant), respectively.

The *stroma* population was estimated using the union of FB, MS, and EN cells in Drop-seq data. The Pearson product-moment correlation with the percentage of cells that stained for vimentin was negative (−0.45, Supplementary Figure S3H, middle panel) with a non-significant *p*-value and may be caused by the epithelial cells undergoing EMT (we observed a consistently smaller percentage of the stromal subpopulation compared to the VIM-stained percentage). The CK7 percentage was positively correlated (Pearson’s correlation of 0.24, Supplementary Figure S3H, right panel) with the *epithelia* (EP and ES cells), however not significantly (*p*-value = 0.34). Out of the 18 samples, only 3 samples had more than 30% difference between the stained CK7 and annotated epithelial sub-population.

As seen previously (Olalekan et al., 2021), we noted significant differences in the abundance of T cells between samples reported by Drop-seq. The T cell percentages in Drop-seq data showed the highest correlation with CD45 staining in IHC. Due to the correlation between T cells in tumors and cancer outcome (Olalekan et al., 2021), we categorized a sample as having high T cell infiltration (T_inf_) if the percentage of T cells was greater than 10%, and low T_inf_ if less than 10% in the sample as per Drop-seq data (Supplementary Figure S2A; Table 2).

### 2.6 Inferring cellular interactions in the tumor microenvironment using ligand-receptor analysis

**FIGURE 4:**
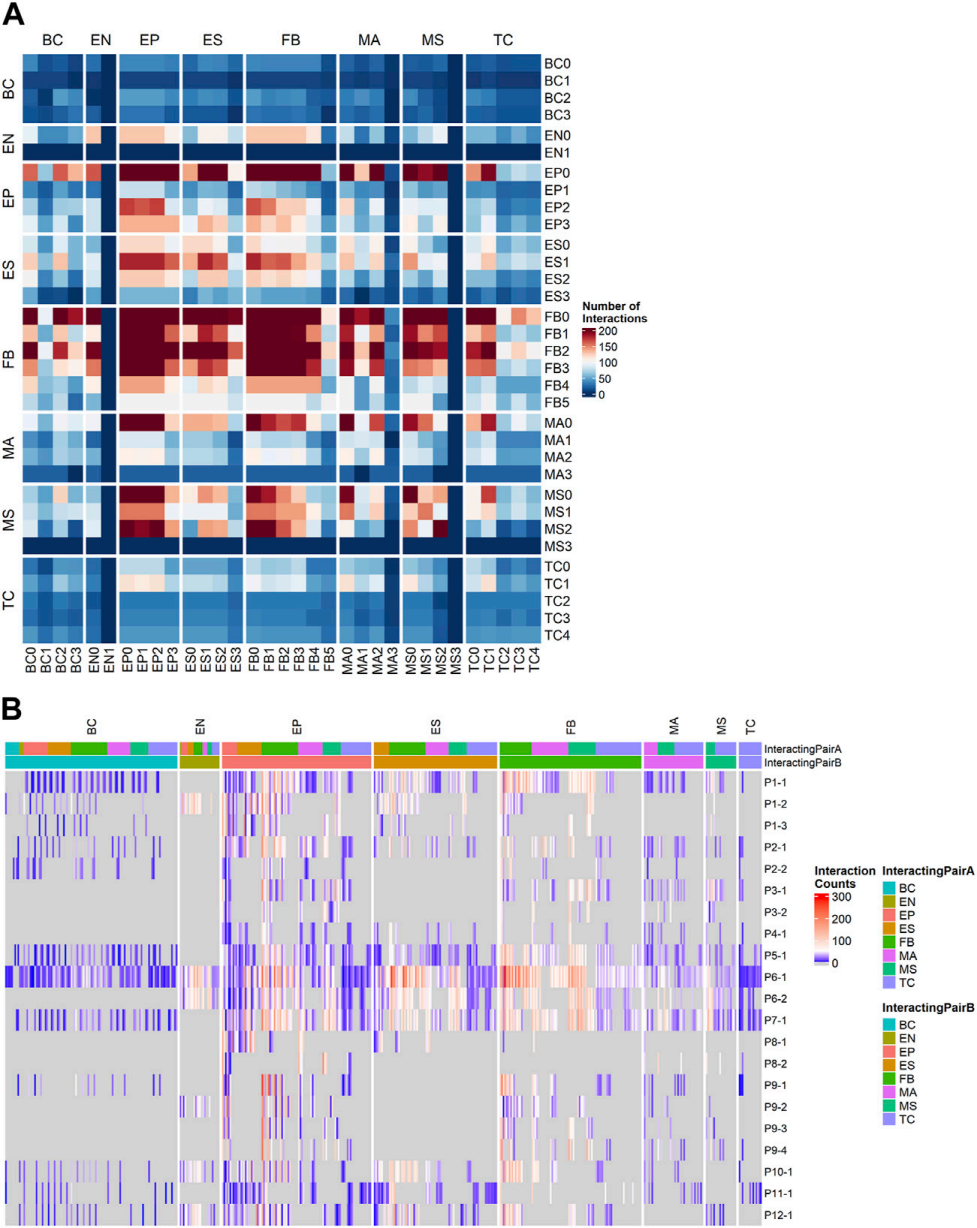
Ligand-receptor (LR) interactions predicted by CellPhoneDB using a customized cancer database. **(A)** Total number of interactions between all cell subtypes. **(B)** Counts of significant Ligand-receptor pairs for all cell type subclusters stratified by sample, the columns are grouped by the cell lineage of the first interactor.

To understand the patient-specific TME, we predicted the ligand-receptor interactions among the cell sub-clusters, using *CellPhoneDB* (Efremova et al., 2020) and additional cancer-specific ligand-receptor (LR) pairs that were curated from previous studies (see Section 4). We found that FB, EP, ES, and MS cells were highly activated for ligand-receptor (LR) interactions (Figure 4A). The higher abundance of FB and EP cells in the TME and high numbers of putative LR interactions identified within and between EP, FB, and immune cells in our data allowed us to further dissect histotype- or site-specific LR repertoires. Accordingly, we selected the following lineage pairs: epithelial-to-fibroblast, immune-to-epithelial, and immune-to-fibroblast. Clusters with less than 50 cells were excluded from the downstream LR analysis.

We first examined the resulting cancer-specific LR interactions in epithelial-to-fibroblasts. To identify LR interactions common to each histotype, we integrated interactions from all samples grouped by their histotypes. Histotype-specific LR signatures across epithelial-to-fibroblasts were identified (Supplementary Figure S4A). HGSOC displayed higher interactions of receptors *ITGB1* in epithelial cells (to *COL1A2*, *MDK*, and *VEGFA* in fibroblasts), as well as *FGFR1* in epithelial cells (to *FGF12* and *FGF18* in fibroblasts). LGSOC histotype had higher LR signatures for *ITGA5*_*ADAM17*, *MET_SEMA5A*, *LAMB1*_*ITGA2*, *LAMC1*_*ITGB4*, and VEGFA_NRP2. Receptor *FGFR1* was also highly expressed in epithelial cells in LGSOC, though the ligand it enriched for was *FGF9*. Clear cell histotype has unique signatures of *CCL2_CCR3*, *SILT2_SDC1*, *BMP2_BMPR2*, and *FBN1_ITGA5*. The endometrioid histotype displayed receptor FGFR3 in fibroblast, and ligands *HSP90AA1* and *FGF12* in epithelial cells. MMMT histotype showed ligand *IGF2* in epithelial cells and receptors *INSR*, *IGF1R*, and *IGF2R* in fibroblasts. Histotype with SOC features (patient P7 only) shared some LR signatures with HGSOC and LGSOC while having distinct combinations of *IGF1_IGF1R*, *SLIT2_ROBO1*, and *EFNA5_EPHB6* (Supplementary Figure S4A).

For cancer associated LR interactions from the immune-to-epithelial (Supplementary Figure S4B), the HGSOC patients had higher *ITGA4*_*MDK* and *BTLA* (to *VTCN1* and *TNFRSF14*). LGSOC was enriched for *THBS1* (interacting with *CD47*, *ITGA3*, *ITGA6*), and *ITGB1* (interacting with *LAMC2*, *ADAM17*, and *TGFBR2*). The SOC histotype predicted *CD44* binding to *VIM* and *FN1.* Clear cell histotype had signatures of *CCL5*_*CCR3*, *ITGA4*_*VCAN*, *CD44*_*SPP1*, *KLRD_HLA-E*, and *IL2RB*_*IL15.* Endometrioid histotype had distinct signatures, such as *C1QB*_*LRP1,* and *TNF*_*LTBR*. The LR pairs for MMMT histotypes were *MMP9_LRP1, LRP1* (to *PSAP*, *SERPING1*, and *A2M*), *COL2A1_DDR1* and *ITGB1_COL2A* (Supplementary Figure S4B).

The cancer-associated LR interactions in immune-to-fibroblast subset (Supplementary Figure S4C) identified high number of *CD44* and *VIM* receptors interacting with *COL1A1* and *ITGB1_COL1A2* for the HGSOC histotype. LGSOC has higher *AREG_EGFR*, *INSR_NAMPT*, *EREG*, and *HBEGF* to *EGFR*, *TFGB1_TFGBR3* interactions, and shared *ITGA4_THBS1* interaction signatures with HGSOC. SOC shared *ITGB1* (to *THBS2* and *LAMB1*) interactions with HGSOC. Signatures of *ITGA6* interactions with *THBS2* and *LAMB1* were higher in SOC histotype alone. *DDR2_COL1A1*, *IGF2R_IGF, VEGFB_NRP1*, and *PDGFA*_*PDFGRA* were exclusively present in the MMMT histotype. Endometrioid histotype also had unique signatures, such as *CD44_LAMC3, PTPRC_CD22*, and *FN1* (with *ITGA8* and *ITGA9*). The *KLRD1_HLA-E* and *ITGB7_VCAM1* were found in the clear cell histotype (Supplementary Figure S4C).

For samples with abundant immune subpopulations, it is feasible for us to break down the immune cells into those compartments with sufficient number of cells captured. The original *CellPhoneDB* database was used to capture commonly occurring LRs that may not be specific to cancer in immune cell subpopulations. We ranked samples by the number of LR interactions (Figure 4B) and selected four samples with high LR interactions for comparison: P1-1 (metastatic, low T_Inf_), P6-1, P7-1 (metastatic, high T_Inf_), and P5-1 (primary, high T_Inf_). In particular, we examined LR interactions of T cells with fibroblasts (Supplementary Figure S5A) and ESC (Supplementary Figure S5B). We observed common signals for *TIGIT* in T cells and *NECTIN2* in fibroblasts and ESC; *TIGIT* contains ITIM motifs in its cytoplasmic tail that binds to *NECTIN2* and triggers inhibitory signals (Deuss et al., 2017). This ligand-receptor signal was lower in P5-1 (Supplementary Figures S5A, B), which came from the primary tumor site. Similarly, the *IL7R_IL7* pair was observed in all four samples for fibroblast (Supplementary Figure S5A), with the lowest signal in P5-1 (*IL7R_IL7* was observed in P1-1 and P6-1 for ESCs, see Supplementary Figure S5B). This ligand-receptor pair has been correlated with immune cell infiltration in the TME (Wang et al., 2022). The ligand *FASLG* in T cells to receptors *FAS*, *TNFRSF10A*, and *TNFRSF1B* in fibroblast interaction pairs were detected in all samples (P5-1, 6-1, and 7-1) but not in P1-1 (Supplementary Figure S5A). Their interaction leads to apoptosis of thymocytes that fail to rearrange correctly their T cell receptor (TCR) genes and activation-induced cell death responsible for the peripheral deletion of activated T cells (Volpe et al., 2016). For fibroblast interactions, the *LGALS9*_*CD44/r* was enriched in both fibroblasts and ESC in P6-1 and P7-1, which are metastatic, with high T_inf_ (Supplementary Figures S5A, B). The *LGALS9*_*CD44* pair appeared on ESC from all samples except P6-1 (Supplementary Figure S5B). Gal-9 has direct cytotoxic effects, binds to CD44 expressed on cancer cells to limit cancer metastasis, and enhances the stability and function of adaptive regulatory T cells (Wu et al., 2014; Ustyanovska Avtenyuk et al., 2021). Different interactions associated with immune regulation in tumors (Li et al., 2021) were also found: *CD74* interactions with *APP* or *COPA* were found in samples P5-1 and P6-1, while *HLA-C_FAM3C* interaction was enriched in samples P1-1 and P7-1 (Supplementary Figures S5A, B). *CD2_CD58* interactions between T cells and ESC were noted in all four samples (Supplementary Figure S5B), and between T cells and fibroblasts in P6-1 and P7-1 (Supplementary Figure S5A). *NOTCH2* interactions (Galic et al., 2013; Jia et al., 2019) (with *JAG2* and *DLL3*) were seen in fibroblasts in P1-1 (Supplementary Figure S5A).

We also detected intriguing patterns of certain integrin complex-collagen binding pairs (Zeltz and Gullberg, 2016) on fibroblasts enriched in specific samples (Supplementary Figure S5C): integrin complex *A2B1* appeared in P6-1 only; enhanced expression of α2β1 integrins may influence spheroid disaggregation and proteolysis responsible for the peritoneal dissemination of ovarian carcinoma (Shield et al., 2007). *A1B1* was intriguingly absent in P1-1; instead, integrins *A10B1* and *A11B1,* appear in P1-1 alone. Integrin α11β1^62^ was previously seen overexpressed in NSCLC, especially in CAFs (Zhu et al., 2007; Navab et al., 2011). The *CD40LG_A5B1* pair was seen for fibroblasts in P1-1, P6-1, and P7-1 (Supplementary Figure S5A). Integrin α5β1 plays an important role in tumor progression (Hou et al., 2020). In addition, strong *A4B1* interactions with *FN1, VCAM1* and other ligands (Baiula et al., 2019) are seen with fibroblasts in P5-1, P6-1, and P7-1 (Supplementary Figure S5A), and ESC in P6-1 (Supplementary Figure S5B); *A4B1* receptors have been proposed to target therapy in inflammatory disorders and cancer (Baiula et al., 2019).

These results suggest that different patient samples may have unique LR signatures that are associated with specific cell types, which may be used to target therapy.

## 3 Discussion

Ovarian cancer is a collection of different carcinomas that manifest as different histotypes, each with different cellular compositions and pathogenic mechanisms. Analysis of the TME in different ovarian cancer histotypes at the single-cell resolution can potentially connect the different histotypes with their unique cellular and molecular signatures, understand disease etiology, and help guide therapy. With this aim in mind, we ran Drop-seq on 21 tumor samples from 12 patients and across 6 histotypes of ovarian cancer. We detected three major cell compartments: epithelia (epithelial cells and ESC), stroma (fibroblast, endothelial cells, and MSC), and immune (T, B, plasma B, macrophage) by integrating all single-cell experiments. The four ovarian cancer subtypes using the TCGA gene expression signature revealed highly correlated cell types: the immunoreactive subtype showed a higher correlation with immune cells, while the mesenchymal subtype correlated most with stroma cells and least with epithelial cells. The differentiated and proliferative subtypes both consisted of epithelia but with low and high percentages of ESC, respectively. This suggests that the molecular subtypes classified by TCGA may be driven by the cell type compositions of the tumor samples. Because each tumor sample showed a unique cellular makeup that differed between primary and metastatic sites, it follows that the dominant molecular subtype of a tumor sample is specific to its site of origin, rather than being patient-specific, e.g., patients, P6 and P8, while sharing the HGSOC histotype, have different TCGA subtypes.

For most cell types, we found that the cell cycle phases G1/S, G2/M, and M/G1 were consistently distributed with a higher percentage of the M/G1 phase, with the exception of ESC, where over 70% of the ESC belonged to the G2/M phase. Tumors with high G2/M gene activity have been associated with metastasis and worse outcomes in patients with particular subtypes of breast and pancreatic cancers (Oshi et al., 2020a; Oshi et al., 2020b). The role of p53 in G2/M related cell-cycle arrest in response to DNA damage has been studied extensively (Agarwal et al., 1995; Concin et al., 2003; Müller et al., 2020).

We found five different subtypes of cancer-associated fibroblasts, FB0 and FB2-5 in both primary and metastatic sites, based on the expression of *IL6*, *CCL2*, *S100A4*, *PDPN*, and *FGF7*. Each CAF sub-cluster supports different roles in the progression and metastasis of ovarian cancer. Cells in FB0 expressed genes associated with angiogenesis, Integrin signaling, and T cell receptor signaling pathways. These pathways were related to extracellular matrix remodeling and immune crosstalk under the tumor micro-environment (TME). FB2 supported upregulation of NF-kappa B signaling pathway genes and chemokine receptors associated with cancer metastasis. FB2 and FB4 exhibited elevated expressions of growth factor binding genes as well as genes enriched for angiogenesis and blood vessel development. Top differentially expressed genes in FB3 may be involved with endothelial cell signaling and vascular function. FB5 showed genes enriched in immune crosstalk and cytokine/interferon signaling pathways. Among epithelial cells, we identified the EP3 sub-cluster as cancer stem cells, based on high expression of *IFIT1* and *ISG15*.

The majority of the immune sub-clusters were consistent with those identified in our previous study on metastatic ovarian cancer (Olalekan et al., 2021). We identified a new cluster of IL32 + B cells (BC2) that are CD38-SDC1-S100A4+GAPDH+; these cells were found in both primary and metastatic tumor sites with high T cell infiltration, deriving primarily in clear cells and SOC histotypes.

We did not observe any significant difference in the overall composition of cell lineages between primary vs. metastatic sites. We noted higher ratios of specific CAF (FB4 and FB2) and macrophage (MA2 and MA3) subsets and lower ratio of an epithelial subcluster (EP2) in metastatic sites, compared to primary tumors.

Overall percentages of T and B cells were higher in high T_Inf_ samples, be it from primary or metastatic site, while the percentage of MS was lower overall. At the sub-cluster level, the TC0 and BC3 were positively correlated with T_Inf_ status, with MS2 showing a negative correlation. The CAF sub-clusters FB0, FB2, FB4, and FB5, and the CSC (EP3) were enriched in samples classified as metastatic, low T_Inf_.

The IHC and GWAS markers showed distinct expression patterns on different histotypes, especially for the epithelial and stromal lineages. The immune lineage was overall less sensitive to these known markers.

Besides tumor site and T_Inf_ status, there were also differences in the makeup of cellular sub-types between histotypes. The percentages of epithelial cells from EP1-3 were higher in HGSOC and MMMT histotypes, while the percentages of ESC in clusters ES1-3 were higher in HGSOC only. For clear cell histotype, the percentage of cells in TC0, BC2, and BC3 was higher. The endometrioid histotype had a higher percentage of MS and FB1 cells. The percentages of MA0, MA2, and TC1 cells were higher in LGSOC than in other histotypes.

Lastly, we found fibroblasts and MSC to be active players in the TME, exhibiting potentially distinct LR interactions with epithelial and immune subclusters in patients and histotypes. Imputed ligands and receptors may be leveraged to target therapy in ovarian cancer patients.

*Limitations of the study:* The total number of patient samples collected in this study is limited due to the pandemic. Certain cancer subtypes such as MMMT were less represented in our samples because of their lower prevalence (Xu et al., 2020). We observed marked heterogeneity in the patients’ TME in our datasets; however, due to limitations of sample size, we focused on conservative signals within each group of interest. The treatment and outcome information were not available for the patient cohort and therefore, could not be included in the analyses. The cell sub-populations in tumors dissected from different individuals, tumor sites (primary vs. metastatic) or even different regions sampled from the same tumor may vary. The ligand-receptor interactions were inferred *in silico* through statistical testing, with the caveat that the same ligand or receptor can account for multiple inferred ligand-receptor pairs. Further validation tests are needed to confirm the ligand-receptor interactions.

## 4 Materials and methods

### 4.1 Tissue collection, sample preparation, and drop-seq

Ovarian cancer tissues from primary and metastatic sites were collected from women undergoing debulking surgery at the University of Chicago. Some of the tissue collected from the different sites were patient-matched. The University of Chicago’s Institutional Review Board for human research approved the collection of human tissue after patient deidentification. Ovarian tumors were transported in DMEM/F12 containing 10% FBS and 1% P/S (100% DMEMF/12), and processed as previously described (Olalekan et al., 2021). Red blood cells and dead cells were removed from cell suspensions using Miltenyi Biotec, 130-094-183, 130-090-101, respectively, used according to manufacturer’s protocols. Additionally, some samples were enriched for immune-only, non-immune, tumor-only, and non-tumor cell compartments, using magnetic bead-based isolation or fluorescence activated flow sorting (Miltenyi Biotec, 130-118-780, 130-045-801, 130-108-339, 130-042-401, 130-112-931, 130-118-497, and 130-110-770, used according to manufacturer’s protocols).

Drop-seq was performed as previously described (Olalekan et al., 2021) on ovarian cancer tumor samples from 12 patients (Table 1). A total of 21 tumor samples were present in this study, including 5 patients with Matched primary (right and/or left ovaries) and metastatic (omentum, rectum) tumors (Table 2). Of these, a few randomly selected samples were enriched for select cellular compartments prior to running Drop-seq: CD45^+^ (5 samples), tumor (2 samples), and non-tumor (1 sample); 18 samples were processed without any enrichment.

### 4.2 Data processing, alignment, and clustering analysis

A total of 40 sequencing runs were performed on Illumina’s NextSeq 500 using the 75 cycle v3 kit, as previously described (Olalekan et al., 2021). Some samples were sequenced multiple times to achieve deeper resolution. Each run produced paired-end reads, with Read 1 representing the 12 bp cell barcode and a 6 bp long unique molecular identifier (UMI), and Read 2 representing a 60–64 bp mRNA fragment. Paired-end reads from the same samples were combined to generate 26 paired-end fastq files. Read count matrices were generated from sequence reads from the Drop-seq experiments for both exon and intron regions in the human genome (gencode (Frankish et al., 2019) hg38 v.27) using a *Snakemake* pipeline (Selewa et al., 2020) and STAR version 2.5.3 aligner (Dobin et al., 2013).

To select high-quality cells, we applied a filter based on the number of genes detected per cell. Prior to filtering, each sequenced sample produced approximately 5,000 cells. Based on the median number of captured genes per cell, cells with less than 400 genes detected were removed from the dataset. A total of 26,421 cells were retained for the downstream analysis. We followed a standardized pipeline using the single-cell analysis tool suite, Seurat v3.0.2 (Butler et al., 2018; Stuart et al., 2019). A logarithmic normalization method (Butler et al., 2018) was applied to all samples to transfer the gene expression counts [+1, to avoid log(0)] scaled by a factor of 10,000 (TP10K) to log units. The normalized matrices for all samples were integrated by the anchor-based alignment method Canonical Correlation Analysis (CCA) using Seurat (Stuart et al., 2019). The top 1,311 highly variable genes and top 20 canonical vectors were selected to perform the alignment integration, where the integrated gene expression matrix had a lesser number of features (genes) than the original gene expression matrix. The integrated matrix was scaled by a linear transformation to center the mean gene expression for all cells. We applied PCA on the scaled integrated expression matrix to extract the top 50 components in the data, followed by a heuristic elbow plot on the standard deviation of each PC. We selected the top 16 variant PCs based on the elbow plot. The selected PCs were used in further exploration of the data, such as UMAP (McInnes et al., 2018) dimension reduction, construction of K-nearest neighbor graphs, shared nearest neighbor modularity optimization-based clustering (Stuart et al., 2019), etc. We used dimension reduction methods, UMAP, to generate 2D plots to visualize different cell populations in the experiments. Hierarchical clustering on the shared nearest neighbor graph was applied to infer the clustering structure on the cells where the resolution parameter was set to 0.2. Differential expression analysis was performed through the *FindMarkers* function in Seurat using the Wilcoxon Rank Sum test, and statistically significant markers were extracted for sub-populations or contrast groups based on an adjusted *p*-value (adj. p-val.) threshold of 0.05.

### 4.3 Cell cycling effects

We inferred the cell-cycle phase for all cells based on previously curated gene markers reflecting three phases of the cell cycle in chemically synchronized cells (G1/S, G2/M, and M/G1) (Whitfield et al., 2002; Evan et al., 2015). For each cell-cycle phase, the module scores were calculated as the average expression levels of binned gene markers subtracted by the aggregated expression of random gene sets from the same bin. The Seurat *AddModuleScore* function was used to assign all five module scores to each cell where 24 bins of aggregate expression levels for the marker genes were used and a hundred control genes were selected from the same bin per gene. The highest scored cell-cycle phase was assigned to the cell. If none of the module scores were positive, the cell was designated as not assigned (NA).

### 4.4 Cancer subtype classification

Four cancer subtypes-differentiated, immunoreactive, mesenchymal, and proliferative were categorized by previous bulk sequencing study in ovarian cancer (Tothill et al., 2008; Network, 2011). The marker genes for each subtype were determined by the upregulated marker signatures on the four subtypes (Verhaak et al., 2012). The Seurat *AddModuleScore* function was used to assign four module scores to each cell where 24 bins of aggregate expression levels for the marker genes were used and a hundred control genes were selected from the same bin per gene. The subtypes were then assigned to individual cells by the highest positive modular score. In the absence of positive modular scores, the subtype was considered not assigned (NA).

### 4.5 Cancer patient survival prediction

The cancer outcome was categorized as poor and good in the previous research on the TCGA ovarian cancer dataset (Tothill et al., 2008; Network, 2011), where a list of gene signatures based on RNA-seq data was extracted for both outcomes. We obtained the module scores based on these lists of predictive gene markers using the Seurat *AddModuleScore* function as described in the cancer subtype classification. The predicted outcome was assigned to the cells according to the module score.

### 4.6 Cell type classification using template-based method

We assigned the cell type using a template-based cell annotation method, namely, sc-TACA (https://github.com/bingqing-Xie/taca) (Xie, 2021). The sc-TACA method utilizes an annotated single-cell dataset as a template. In this study, six HGSOC metastatic samples in the 26 samples have been previously annotated, which was used as the template. The cell types annotated in this template were denoted by $T=\left\{t_{i},t=1..p\right\}$ , where p is the total number of unique cell types. All samples were integrated by an anchor-based alignment via Canonical Correlation Analysis (CCA) in Seurat (Butler et al., 2018; Stuart et al., 2019). Then modularity optimization-based hierarchical clustering *FindClusters* was applied on the integrated dataset with a resolution r = 0.2 that resulted in 11 cell clusters. For each cluster i , we obtained the annotated cell type vector $C_{i}=\left\{c_{1},c_{2},\ldots ,c_{N_{i}}\right\}$ where $N_{i}$ is the total number of cells from cluster i and $c_{i}\in T$ . The annotation $t_{i}$ of a given cluster i was determined by the highest ratio of annotated cell type within the cluster $t_{i}=\underset{t}{\mathrm{argmax}}r_{t}^{i}$ where $r_{t}^{i}=\frac{\sum c_{i}|c_{i}=t}{N_{i}}$ . A threshold $r_{\min}=0.7$ was enforced to ensure the robust assignment. If $\max_{t}r_{t}^{i}<r_{\min}$ for cluster i , it was labeled as undecided.

### 4.7 Immunohistochemistry

Ovarian cancer tissues were fixed and stained for Immunohistochemistry as previously described (Olalekan et al., 2021), to evaluate the fraction of cytokeratin-7 (Thermo Scientific, MA5-11986), pan-vimentin (Abcam, Ab16700), CD45 (Agilent, M0701) positive cells. Aperio ImageScope v12.4.3 was used to analyze the fraction of cells that stained for CD45, vimentin, and CK7 in the entire tissue section, using algorithm “Positive Pixel Count v9”.

### 4.8 Analysis of fibroblasts, epithelial cells, and immune sub-population

After identifying the cell types, we extracted the fibroblasts, epithelial cells, and immune cells (T cells, B cells, macrophages) to conduct further investigation. Each sub-population expression matrix was a subset of the integrated matrix. The expression matrix was scaled and PCA analysis was performed to extract the top components in the data. Top PCs were selected based on the elbow plot, which varied from 10 to 20 based on the sub-population variation. Hierarchical clustering on the shared nearest neighbor graph was applied where the resolution parameter was set to a range between 0.2 and 0.5. The same UMAP was used to project the cells to a 2D space to visualize the sub-types for each cell type. Differential expression analysis was performed through the *FindMarkers* function in Seurat using the Wilcoxon Rank Sum test, and statistically significant markers were extracted for sub-populations or contrast groups based on an adjusted *p*-value (adj. p-val.) threshold of 0.05. The differences in cell composition ratios between primary and metastatic sites, and between high and low T_inf_ groups were evaluated by two-sided *t*-test with *p*-value estimation.

### 4.9 Ligand-receptor interaction analysis on cell type subclusters

We constructed a customized pan-cancer ligand-receptor (LR) interaction database, using *CellphoneDB* (Efremova et al., 2020) and published cancer studies, including 27 immune checkpoint LR pairs (Pardoll, 2012), 114 interaction pairs between cancer cells and T cells in lung cancer (Chen et al., 2020), 1380 LR pairs in a pan-cancer study (Ghoshdastider et al., 2021), and 216 LR pairs related to ovarian cancer (Castellano et al., 2006). For each sample, we inferred LR interactions among any pair of the cell sub-clusters, $SC=\left\{{sc}_{i}^{j}\right\}$ , where i is the lineage such as EP, and j is the subcluster index, using the pan-cancer LR database. We obtained a *p*-value for the likelihood of cell-type enrichment of each ligand–receptor complex ( $L=\left\{l_{i}^{j}\right\}$ , where i is a ligand and j is a corresponding receptor). We denote $\left\{{sc}_{i1}^{j1},{sc}_{i2}^{j2}\right\}$ for a sub-clusters pair. *p*-value is calculated by the proportion of means that are as high as (or higher) than the random permutation for all pairs, $SC=\left\{\left({sc}_{i1}^{j1},{sc}_{i2}^{j2}\right)\right\}$ . Interactions with adjusted *p*-value <0.05 were considered significant. The “significant means” vector, $M=\left\{m_{l}^{sc},l\in L,sc\in SC\right\}$ was extracted for each sample and $m_{l}$ was set to 0 when *p*-value >0.05 or sub-clusters with insufficient cell counts, $\left(\left|sc\right|\right)\;<50$ . The number of absolute interactions $\sum \left(M>0\right)$ was used as a proxy to estimate the frequency of the cell-cell crosstalk among cell types. The hierarchical clustered heatmap was used to identify the shared patterns for the sub-clusters from different cell types. We then grouped the samples by histotype and site for the downstream comparative analysis. A linear model was built using *lmfit* in Limma R library for a given contrast group (e.g., one histotype against the rest of the histotypes), and the empirical Bayes moderated t-statistics test *ebayes* was used to estimate the significance of any LR signature, l∈L (Dixon, 2003; Smyth, 2005). Significant positive LR pairs were used as the signature for any given condition group.

## Data Availability

The datasets presented in this study can be found in online repositories. The names of the repository/repositories and accession number(s) can be found below: https://www.ncbi.nlm.nih.gov/geo/, GSE235931.

## Supplementary material

SUPPLEMENTARY FIGURE S1 Stacked bar plot for cell-type annotations for samples together. **(A)** Cell type composition per sample, grouped by patient. **(B)** Composition of the cancer Genome Atlas (TCGA) molecular subtype per sample, grouped by patient. **(C)** Cell-cycle phase composition by major cell types.

SUPPLEMENTARY FIGURE S2 Immune, epithelial, and stroma lineages shown in Figure 2, with selected markers for each cluster. The cluster number and color are consistent with Figure 2. The expression in the dot plot is the averaged scaled log normalized TP10k value. **(A)** UMAP (top) of immune cells only, and heatmap (bottom) of immune percentages for all samples with unsupervised dendrogram forming the T cell infiltration high/low sub-groups. **(B)** Dot plot of immune cell marker expressions for each immune (TC, BC, MA) subcluster. **(C)** Dot plot of CSC and epithelial cell marker expressions for each EP subcluster. **(D)** Dot plot of ESC, CSC and cell-cycle marker expressions for each ES subcluster. Expression of *TP53* is added for reference. **(E)** Dot plot of CAF and fibroblast marker expressions for FB subclusters. **(F)** Dot plot of MSC marker expressions for MS subclusters. **(G)** Dot plot of endothelial marker expressions for EN subclusters.

SUPPLEMENTARY FIGURE S3 Cell type composition of primary and metastatic tumors of different histotypes and T cell infiltration status. **(A)** Heatmap of cell type subclusters composition percentage for aggregated patient samples by histotype. The column z-scores are the percentages normalized by column. **(B)** Heatmap of cell type subclusters composition percentage for aggregated patient samples by site and T cell infiltration. The column z-scores are the percentages normalized by column. **(C)** Dot plot of histotype markers expression on Immune lineage (TC, BC, and MA) and Stroma lineage (FB, MS, and EN). The expression in the dot plot is the centered log normalized TP10k value. **(D)** Dot plot of GWAS marker expression on Epithelial (EP and ES) and Stroma (FB, MS, and EN) lineages, broken down by histotype. The expression in the dot plot is the centered log normalized TP10k value. **(E)** Dot plot of normal fallopian tube specific, functional, and pan-epithelial markers expression on the epithelial lineage (EP and ES). The expression in the dot plot is the centered log normalized TP10k value. **(F,G)** Immunohistochemical staining for CD45, vimentin, and CK7 are performed on tumor samples of different histotypes and tumor sites. **(F)** Ovarian cancer tumor samples collected from the omentum as the site of metastasis. Six histotypes are shown: malignant mixed Müllerian tumors (MMMT), clear cell, endometrioid with serous features, high grade serous ovarian carcinoma (HGSOC), serous ovarian carcinoma (SOC), and low grade serous ovarian carcinoma (LGSOC). **(G)** Ovarian cancer tumors collected from the ovaries (primary tumor site). Five histotypes are shown: malignant mixed Müllerian tumors (MMMT), clear cell, endometrioid with serous features, high grade serous ovarian carcinoma (HGSOC), and low grade serous ovarian carcinoma (LGSOC). **(H)** The correlations between area staining for CD45, VIM, and CK7 (IHC) with the percentages of immune, stroma and epithelial cells in Drop-seq data, respectively. The gray area shows the confidence interval of 95% from linear fitting. The cell types are abbreviated as follows: EP, Epithelial cells; TC, T cells; MA, Macrophages; EN, Endothelial cells; BC, B cells; FB, Fibroblasts; MS, Mesenchymal stem cells; ES, Embryonic stem cells.

SUPPLEMENTARY FIGURE S4 Ligand-receptor (LR) interactions imputed customized cancer LR database. Significant Means is defined in Methods. **(A)** Between epithelial cells and fibroblasts. **(B)** Between immune cells and epithelial cells. **(C)** Between immune cells and fibroblasts.

SUPPLEMENTARY FIGURE S5 Ligand-receptor (LR) interactions between T cells, fibroblasts, and ESC, imputed using default *CellphoneDB* database. Color bars indicating histotype and T cell infiltration status are the same for all panels. The significant means is defined in the Methods. **(A)** Selected LR interactions between T cells and fibroblasts in four samples. **(B)** Selected LR interactions between T cells and embryonic stem cells (ESC) in four samples. **(C)** Collagen-integrin LR interactions between T cells and fibroblasts in four samples. **(D)** Collagen-integrin LR interactions between T cells and ESC in three samples.

SUPPLEMENTARY TABLE S1
**(A)** Selected cluster markers, and **(B)** enrichment pathways for epithelial sub-populations. **(C)** Selected cluster markers, and **(D)** enrichment pathways for ESC sub-populations.

SUPPLEMENTARY TABLE S2
**(A)** Selected cluster markers, and **(B)** enrichment pathways for fibroblast sub-populations.

SUPPLEMENTARY TABLE S3
**(A)** Selected cluster markers, and **(B)** enrichment pathways for endothelial sub-populations.
