# Supplementary figures and images for: Exploring the tumor micro-environment in primary and metastatic tumors of different ovarian cancer histotypes
Source: Front Cell Dev Biol. 2024 Jan 24;11:1297219. doi: 10.3389/fcell.2023.1297219 (PMC10847324; doi:10.3389/fcell.2023.1297219)

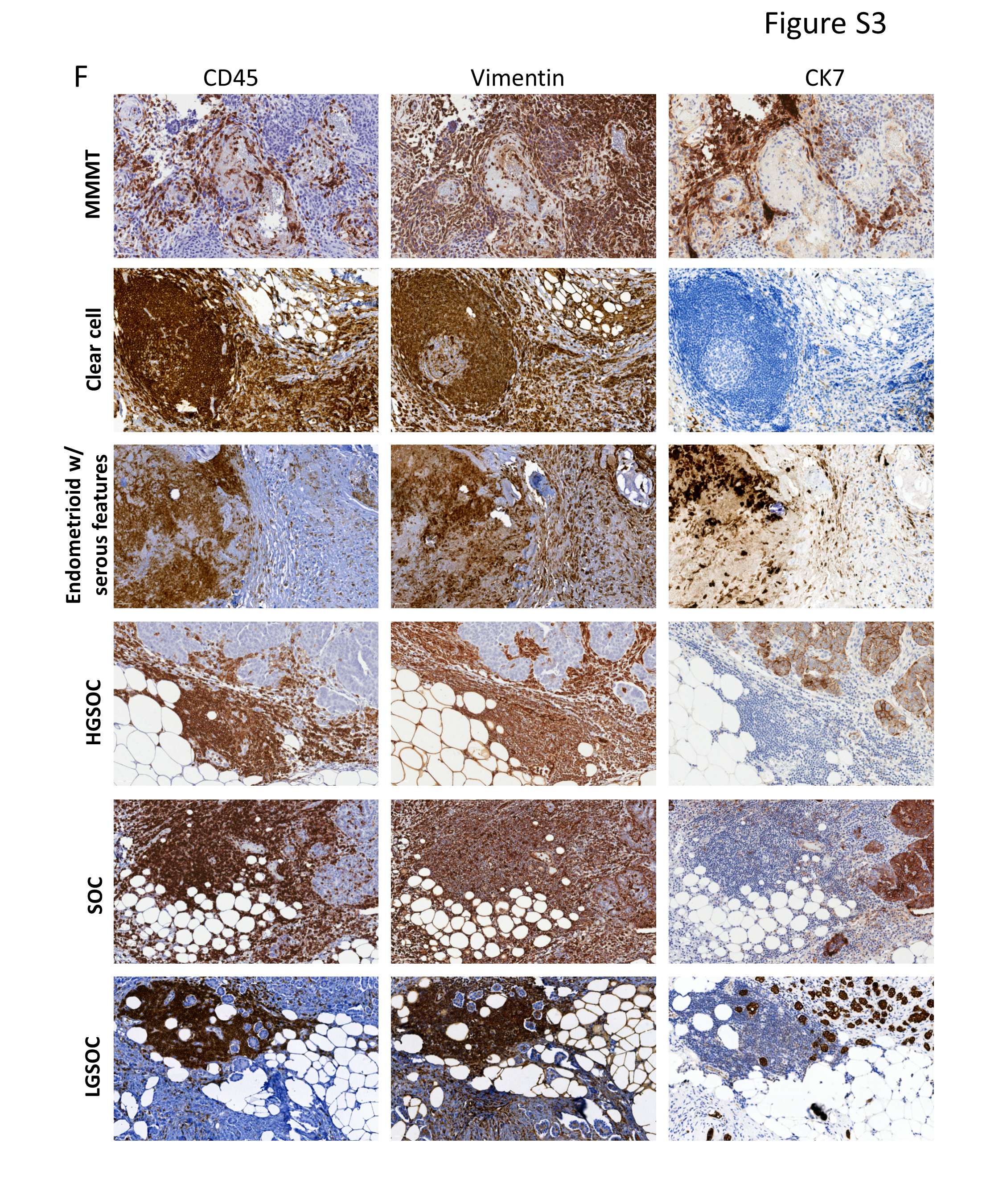

Supplement: Supplementary file 3 [file Image6.TIF]

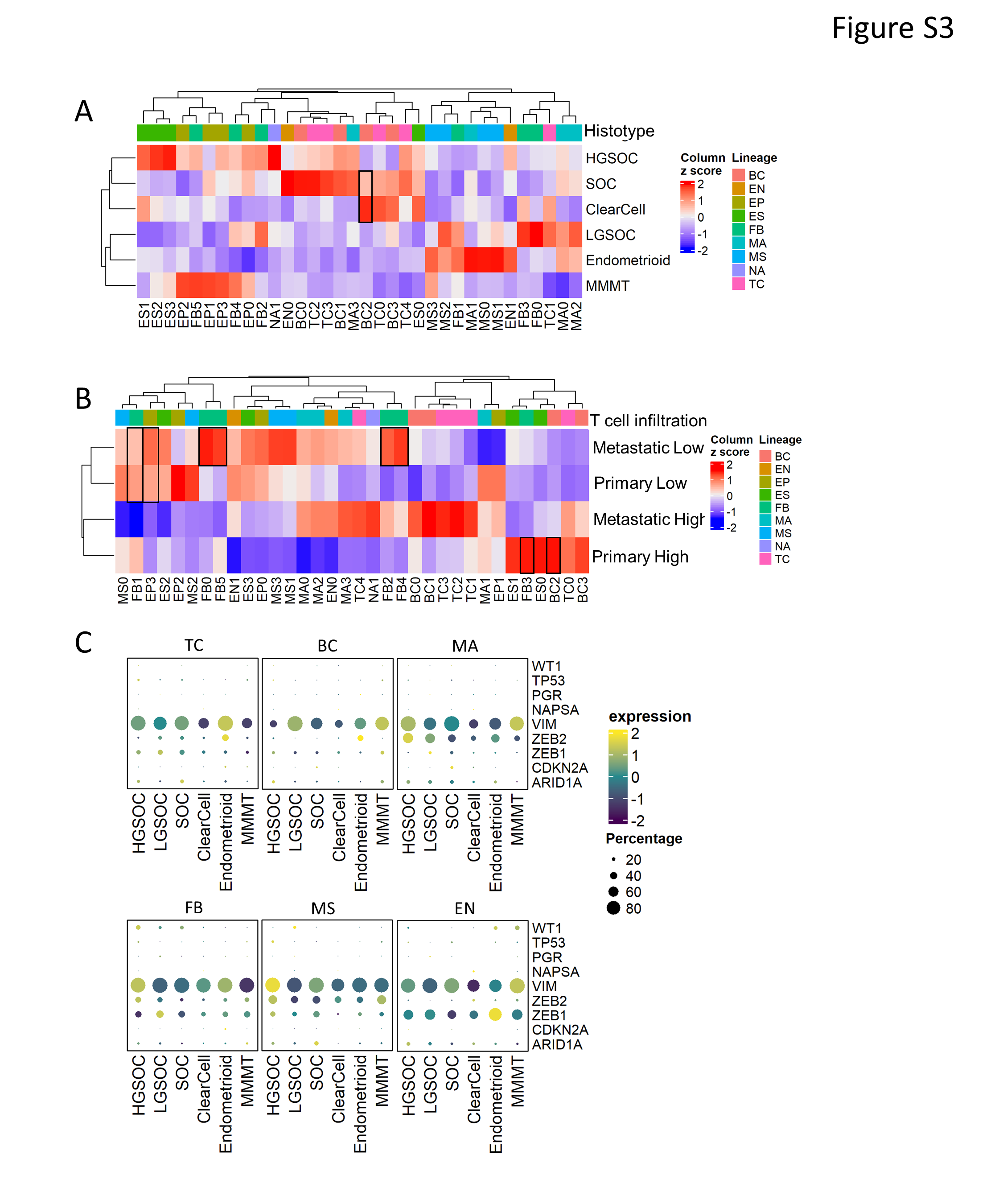

Supplement: Supplementary file 4 [file Image3.TIF]

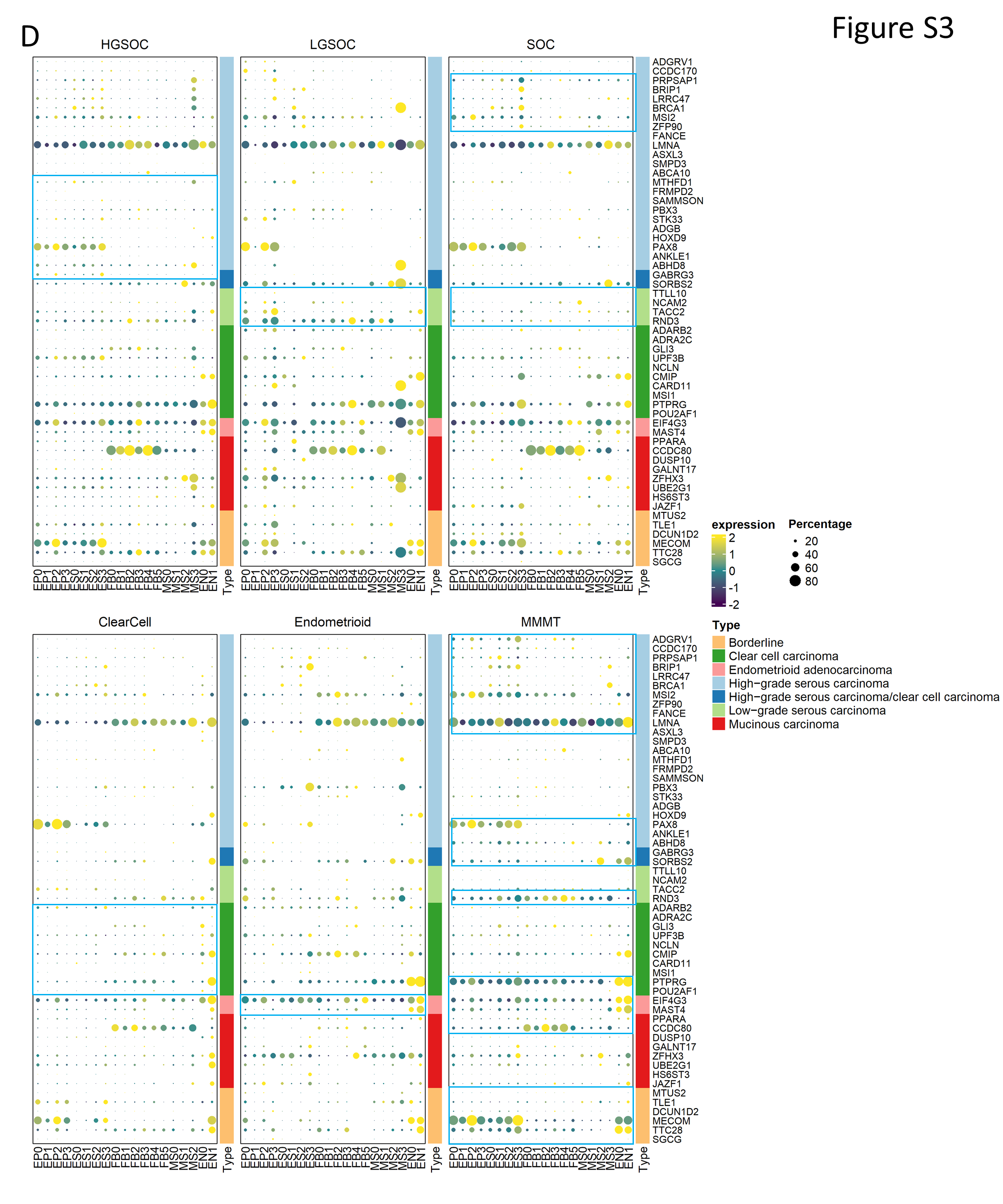

Supplement: Supplementary file 5 [file Image4.TIF]

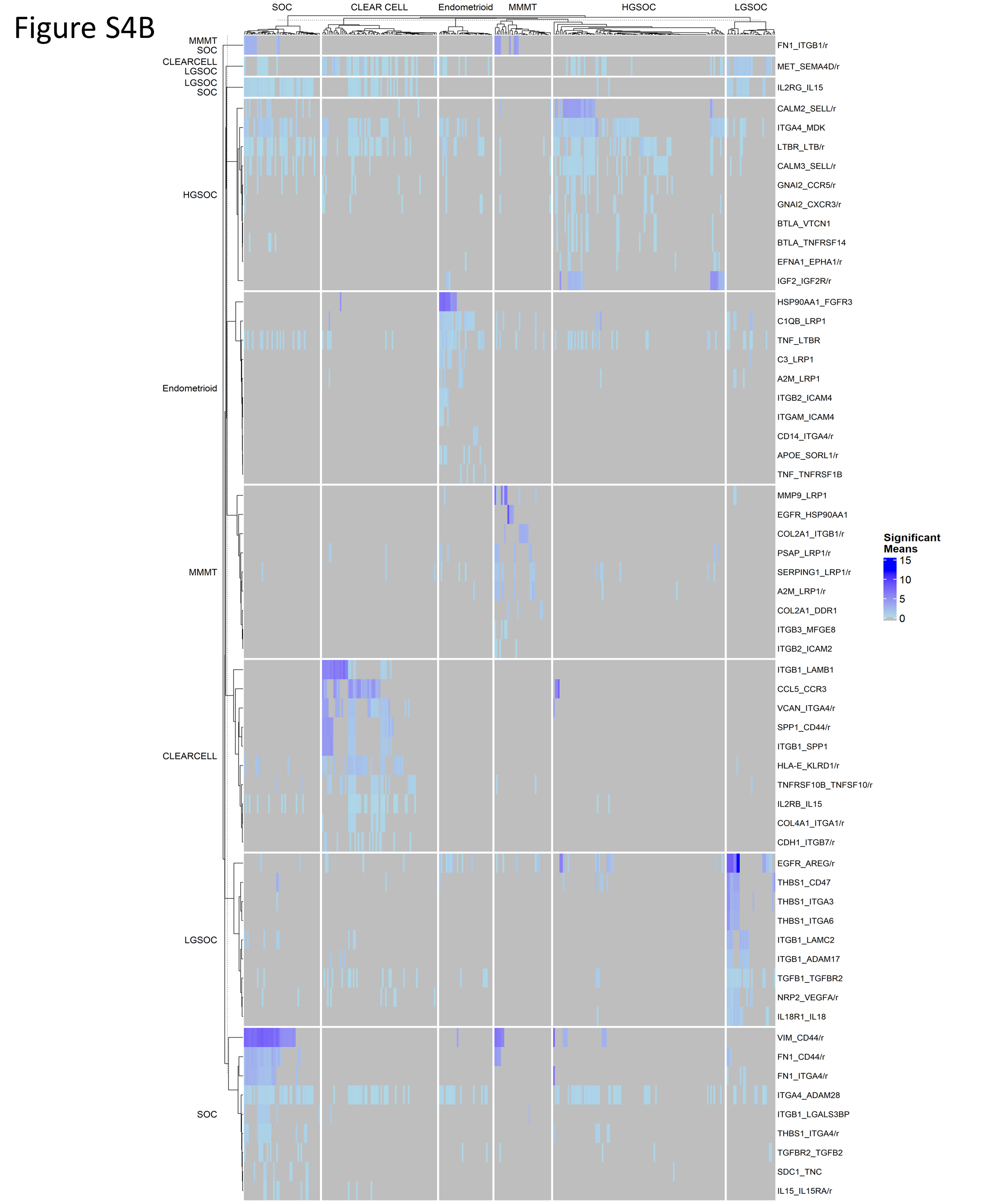

Supplement: Supplementary file 6 [file Image9.TIF]

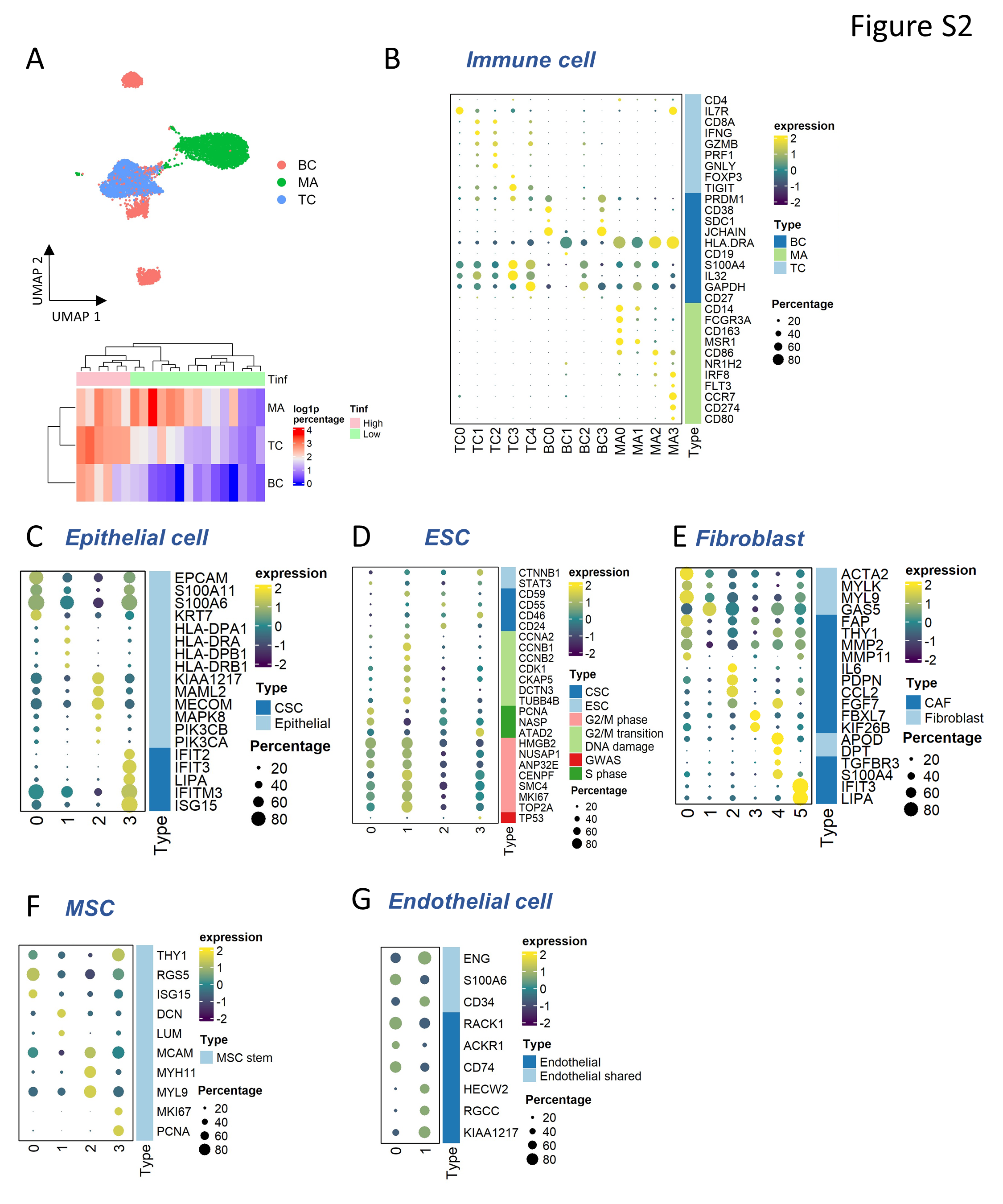

Supplement: Supplementary file 7 [file Image2.TIF]

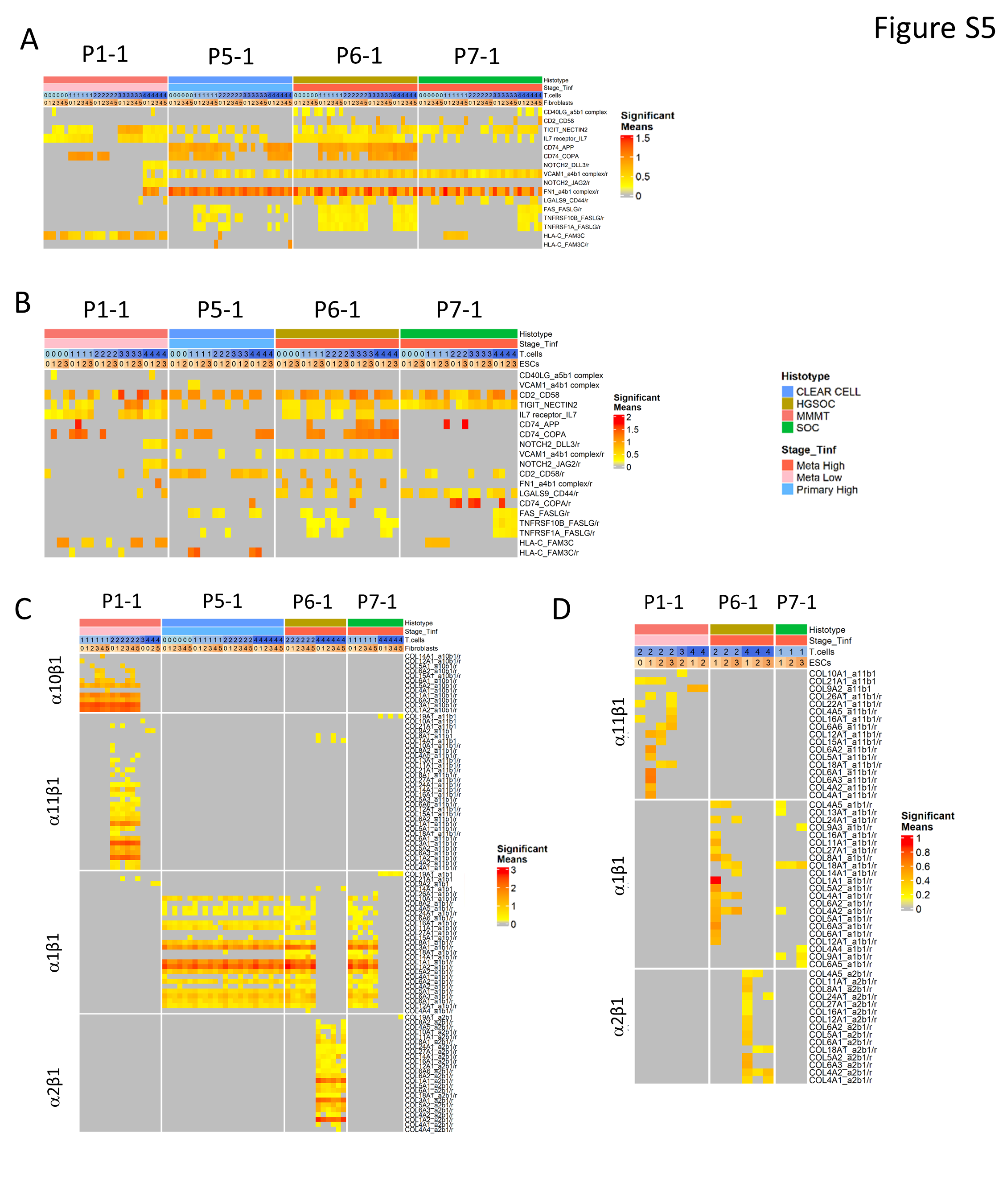

Supplement: Supplementary file 8 [file Image11.TIF]

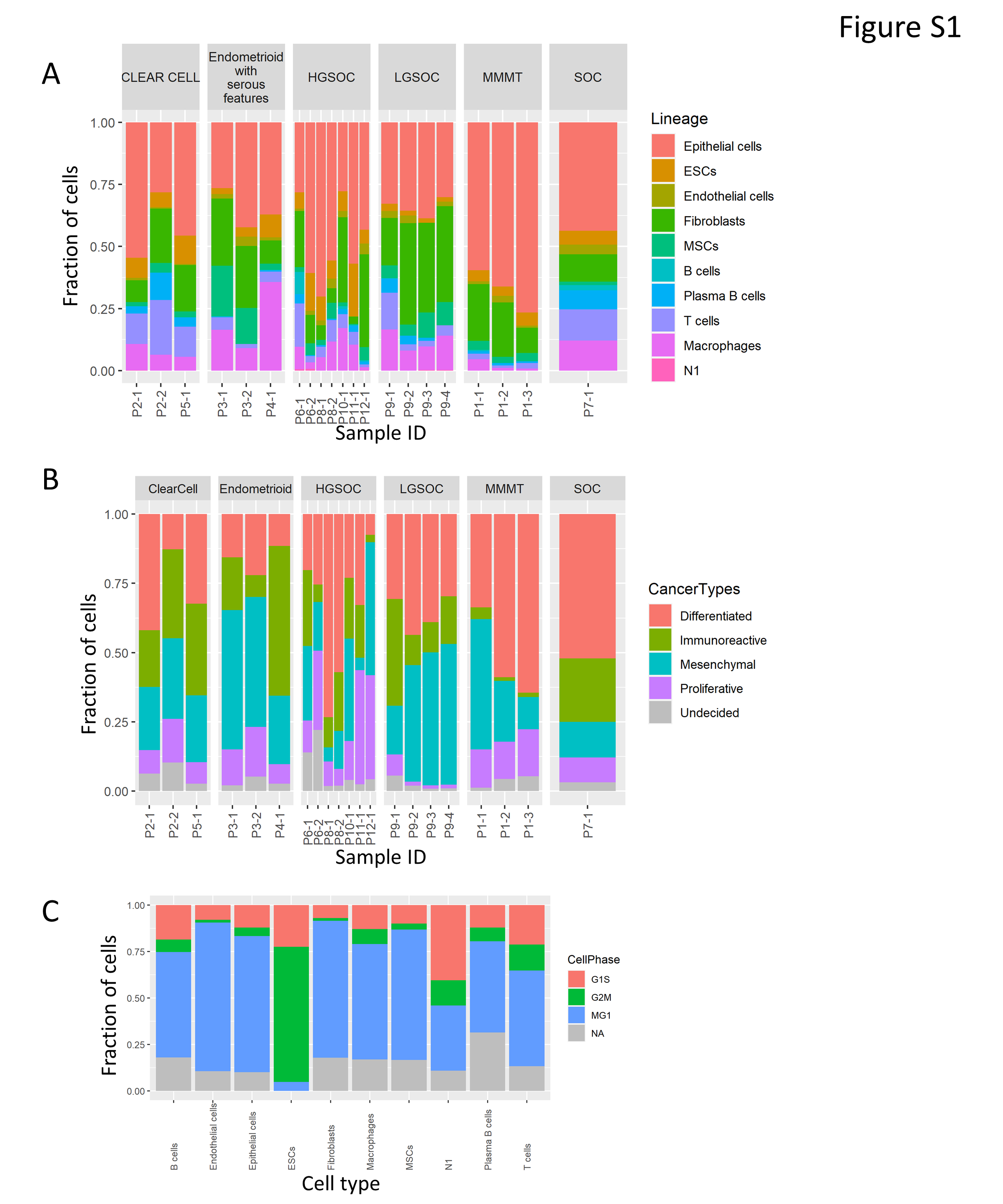

Supplement: Supplementary file 9 [file Image1.TIF]

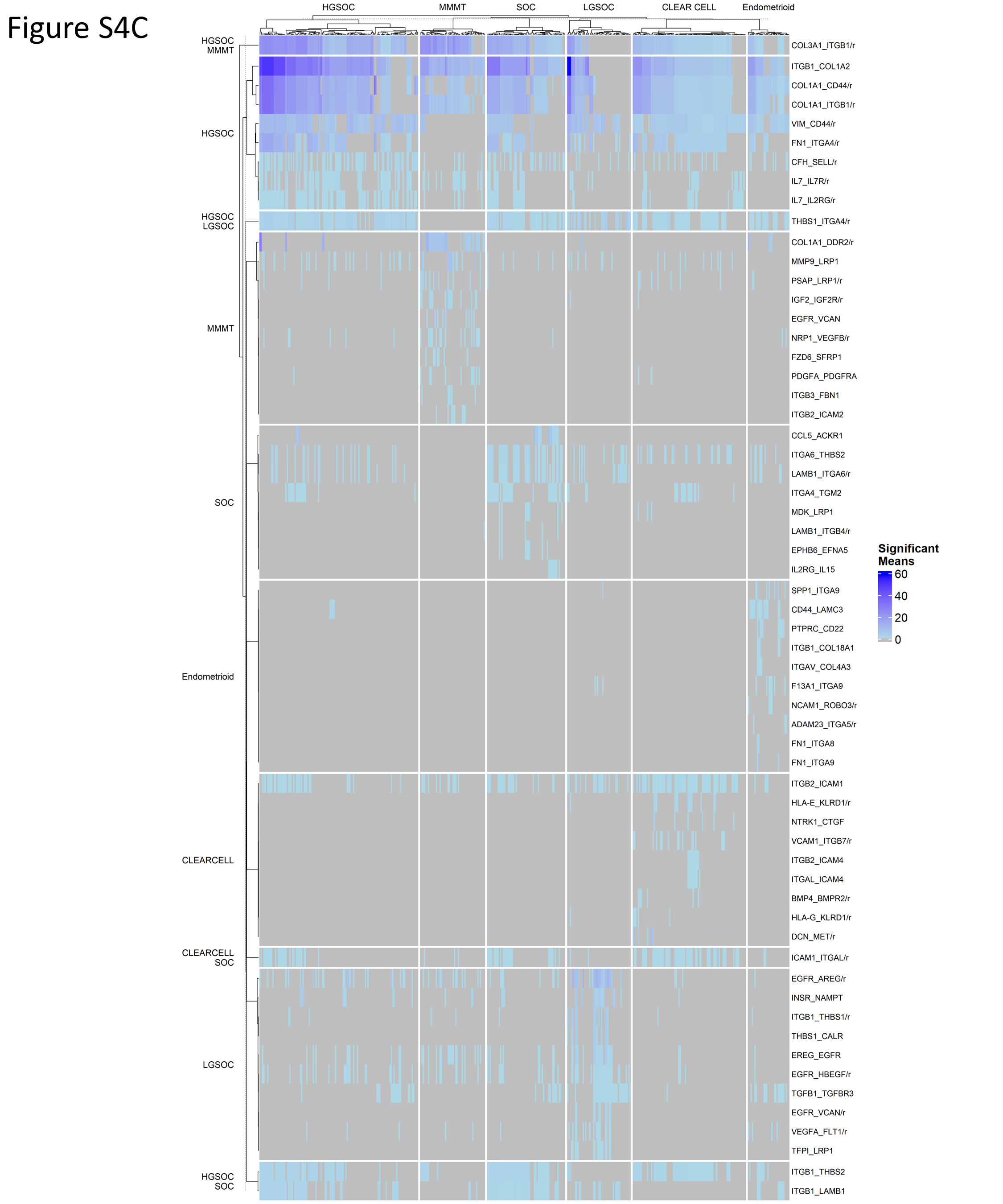

Supplement: Supplementary file 10 [file Image10.TIF]

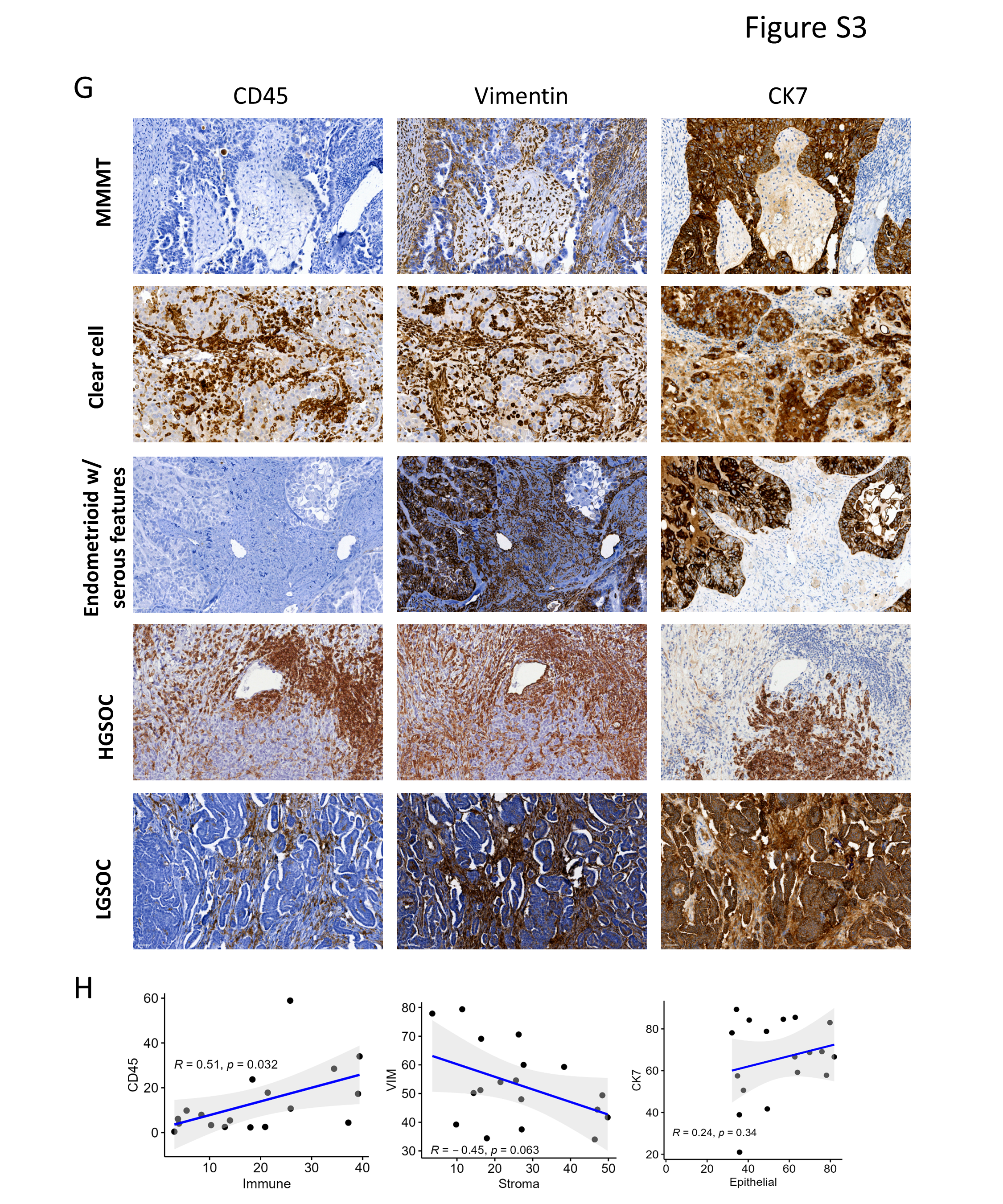

Supplement: Supplementary file 11 [file Image7.TIF]

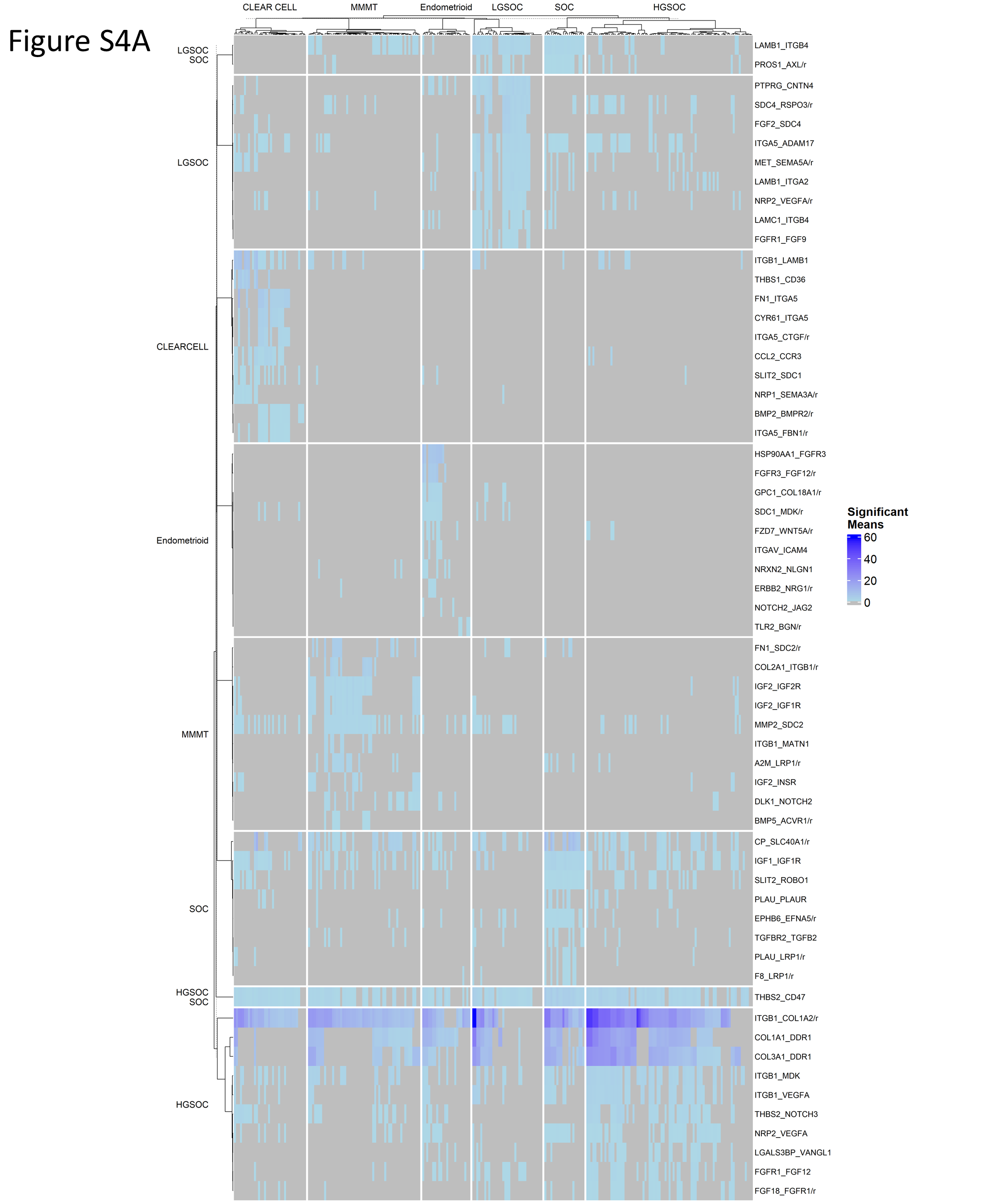

Supplement: Supplementary file 14 [file Image8.TIF]

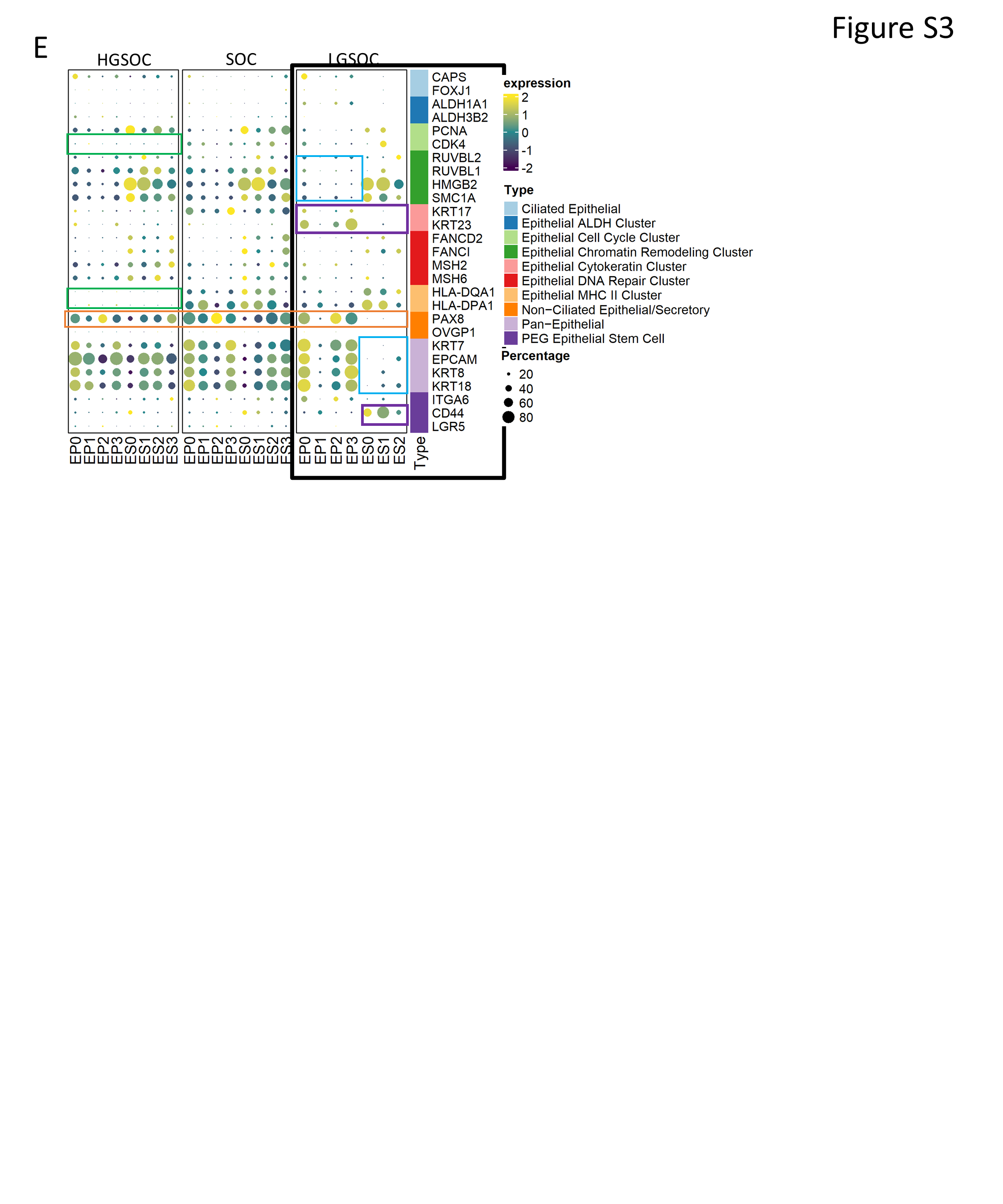

Supplement: Supplementary file 15 [file Image5.TIF]
